# The Single-Nucleotide Resolution Transcriptome of *Pseudomonas aeruginosa* Grown in Body Temperature

**DOI:** 10.1371/journal.ppat.1002945

**Published:** 2012-09-27

**Authors:** Omri Wurtzel, Deborah R. Yoder-Himes, Kook Han, Ajai A. Dandekar, Sarit Edelheit, E. Peter Greenberg, Rotem Sorek, Stephen Lory

**Affiliations:** 1 Department of Molecular Genetics, Weizmann Institute of Science, Rehovot, Israel; 2 Department of Microbiology and Immunobiology, Harvard Medical School, Boston Massachusetts, United States of America; 3 Division of Respiratory Diseases, Boston Children's Hospital, Boston, Massachusetts, United States of America; 4 Department of Microbiology, University of Washington School of Medicine, Seattle, Washington, United States of America; 5 Division of Pulmonary and Critical Care Medicine, University of Washington School of Medicine, Seattle, Washington, United States of America; University of New Mexico, United States of America

## Abstract

One of the hallmarks of opportunistic pathogens is their ability to adjust and respond to a wide range of environmental and host-associated conditions. The human pathogen *Pseudomonas aeruginosa* has an ability to thrive in a variety of hosts and cause a range of acute and chronic infections in individuals with impaired host defenses or cystic fibrosis. Here we report an in-depth transcriptional profiling of this organism when grown at host-related temperatures. Using RNA-seq of samples from *P. aeruginosa* grown at 28°C and 37°C we detected genes preferentially expressed at the body temperature of mammalian hosts, suggesting that they play a role during infection. These temperature-induced genes included the type III secretion system (T3SS) genes and effectors, as well as the genes responsible for phenazines biosynthesis. Using genome-wide transcription start site (TSS) mapping by RNA-seq we were able to accurately define the promoters and *cis*-acting RNA elements of many genes, and uncovered new genes and previously unrecognized non-coding RNAs directly controlled by the LasR quorum sensing regulator. Overall we identified 165 small RNAs and over 380 *cis-*antisense RNAs, some of which predicted to perform regulatory functions, and found that non-coding RNAs are preferentially localized in pathogenicity islands and horizontally transferred regions. Our work identifies regulatory features of *P. aeruginosa* genes whose products play a role in environmental adaption during infection and provides a reference transcriptional landscape for this pathogen.

## Author Summary

Identifying coordinately regulated genes and their control by environmentally-initiated signal transduction pathways is important for understanding bacterial virulence mechanisms. The work reported here provides a comprehensive, high resolution, transcriptome map of the opportunistic pathogen *Pseudomonas aeruginosa* using RNA-seq. The results suggest that *P. aeruginosa* senses the temperature during the transition from its natural environment to a mammalian host, and this plays a key role in regulating the coordinated expression of several virulence factors. A large number of antisense transcripts and non-coding RNAs were identified, with preferential clustering in the regions acquired through horizontal gene transfer, suggesting that a part of the non-coding genome has a distinct evolutionary origin. We created an online data viewer, the *Pseudomonas* transcriptome browser, to facilitate access to the transcriptome data from this study as well as the subsequent results of work deposited by other investigators. The resources generated through our analyses provide a valuable tool to the *P. aeruginosa* research community and set the foundation for a systems biology approach towards understanding the complexity of the regulatory networks controlling the multiple lifestyles of this highly versatile organism.

## Introduction

The outcomes of the broad spectrum of diseases caused by the opportunistic pathogen *Pseudomonas aeruginos*a are determined by several factors related to the predisposing conditions of the infected individual [1], the mechanism of entry [2] and consequences of interaction with the innate defense mechanisms [3]. The versatility of this organism in adapting to a wide range of environments including that of the human body is attributed to a large genomic repertoire consisting of a diverse set of genes encoding metabolic functions suited for proliferation in environments with a wide range of available nutrients [4]–[6]. Moreover, *P. aeruginosa* has an impressive armament of virulence factors, explaining why this species is the only known human pathogen among the members of the genus *Pseudomonas* [7], [8]. Interestingly, virulence genes are highly conserved among different *P. aeruginosa* strains and are part of the core genome, leading to speculations that, in addition to rare infections of humans, some form of pathogenic interaction occurs in natural environments providing the evolutionary pressure for their maintenance [9], [10].

Rapid adaptation of bacteria to changing environments is accompanied by reprogramming of their regulatory networks to activate the expression of genes essential for their survival in the new environment while repressing those that are unnecessary or potentially deleterious [11]. This can be accomplished at the transcriptional level by responding to inputs from various environmental cues that are often mediated by specific signal transduction pathways [12]. Recent advances in high-throughput sequencing approaches allow more accurate quantification of RNA levels in bacteria (RNA-seq) providing significant advances over microarrays [13], [14]. RNA-seq can provide complete coverage of protein-coding genes and intergenic regions to a single nucleotide resolution, and, with adaptation in the library preparation protocols, also allows strand-specific mapping of transcription start sites (TSSs) [15]–[18]. Driven mainly by the data generated from RNA-seq, a number of new regulatory mechanisms were uncovered based on activities of non-coding RNAs [19]. RNA-based regulation (riboregulation) is now recognized as an important mechanism for controlling gene expression by altering the translation of mRNA and/or modulating transcript turnover [20]. Such regulation is performed by *trans*-acting small regulatory RNAs (sRNAs), *cis*-antisense transcripts (asRNAs), and riboswitches. Not unexpectedly, virulence gene expression is also subject to riboregulation [19], [21]–[23].

In addition to small molecule chemical signals, such as nutrients, bacteria also sense and respond to environmental cues, such as the availability of oxygen, altered osmolarity, or pH [24], [25]. Facultative pathogens are also exposed in their natural environments to temperatures that are usually lower than the relatively constant temperature of mammals, 37°C [26]; exposure to this temperature could represent an important signal of environmental change, and trigger expression of specific virulence determinants. Modulation of gene expression by the temperature experienced by microorganisms has been described for a number of facultative pathogens [27], [28]. Moreover, pathogens with a more restricted lifestyle, involving different hosts, can display differential expression of genes depending on the host. For example, a number of proteins encoded on the virulence plasmid of *Yersinia pestis* are expressed at low temperature during growth in fleas, while a different set of proteins is induced at 37°C during the human infection cycle; each set of these proteins is required for growth and survival during a particular stage of infection [29]. Interestingly, bacteria lacking a reservoir outside of the human host can also display temperature-regulated gene expression [30], possibly due to colonization of exposed surfaces such as skin, or during episodes of fever.

Here we present the results of a global transcript analysis using RNA-seq to generate a high resolution map of transcription start sites and identify mRNAs and non-protein coding transcripts (sRNAs and asRNAs) for *P. aeruginosa* PA14. We compare transcript abundance in bacteria grown at the mammalian body temperature (37°C) and an arbitrarily-selected reduced temperature (28°C). Our results present several unexpected findings in regards to temperature and quorum sensing control of gene expression. Moreover, our data provide a useful tool for studies of transcriptional and post-transcriptional regulation of gene expression in *P. aeruginosa* by identifying transcriptional units, accurately mapping transcription start sites and identifying sRNA and asRNA transcripts.

## Results/Discussion

### A number of *P. aeruginosa* virulence genes are regulated by temperature

**Figure 1:**
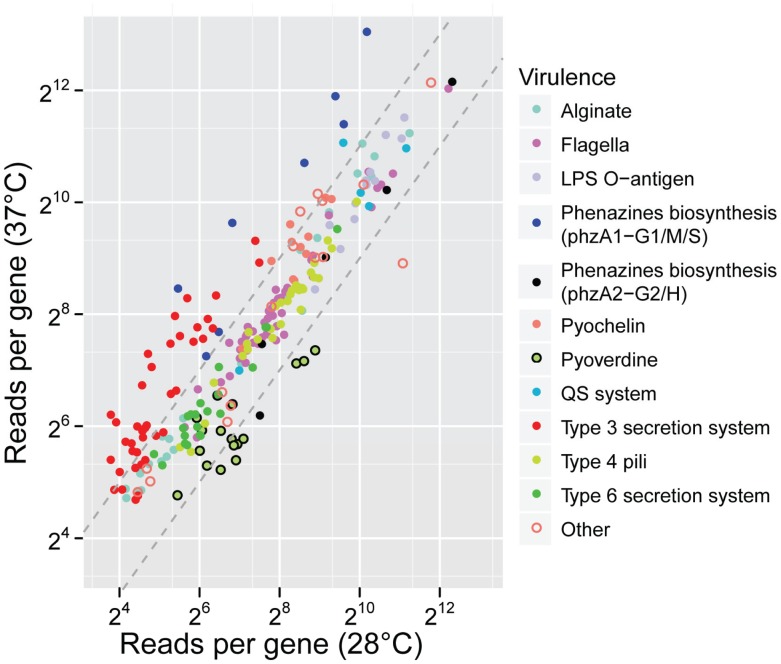
Expression of virulence-related genes at different temperatures. The relative expression of genes known to be related to virulence (downloaded from the Virulence Factors Database [89]) was measured and compared at 28°C and 37°C by RNA-seq. Expression is represented as sequencing read count normalized by gene length and library size for our duplicate samples. Each dot represents a single gene, which is color-coded according to its virulence-related function. The dashed gray lines represent a 2-fold overexpression in 37°C (upper line) and 28°C (lower line) conditions.

We used RNA-seq to examine the global gene expression of *P. aeruginosa* PA14 at 37°C and 28°C, with an objective to assess whether virulence genes respond to alterations in temperatures encountered in different environments (Table S1). Using replicate experiments, we identified a total of 144 genes that showed statistically significant over-expression of transcripts at 37°C, and 234 genes whose mRNA levels were reduced at 37°C compared to 28°C (Table S2). These represent transcripts of genes that may be activated or repressed during infection of warm-blooded mammals and possibly respond to temperature as one of several input signals for their coordinated expression. Therefore, the levels of transcripts for 6.4% of the genes of the *P. aeruginosa* PA14 genome were affected by temperature. Gene ontology (GO) enrichment analysis showed significant enrichment for genes related to protein secretion, phenazine biosynthesis and regulation of alginate biosynthesis among the genes upregulated at 37°C (p = 5.1×10^−6^, 4×10^−3^, and 6×10^−3^ respectively; Materials and Methods). Elevated expression of the regulatory gene cluster (*mucABCD*) leads to repression of alginate production. However, this effect can be overcome in isolates from cystic fibrosis patients in which common mutations in *mucA* lead to overproduction of the alginate exopolysaccharide. At 28°C, we observed upregulation of ribosomal protein genes, histidine catabolic genes, and aromatic and catechol-related genes (p = 6.4×10^−12^, 2.9×10^−3^, 1×10^−3^, and 4×10^−3^, respectively). We found a strong enrichment for virulence-related genes among those upregulated at 37°C (hypergeometric test p = 8.6×10^−15^). Indeed, the expression of 43 out of 238 annotated virulence genes was altered in response to the temperature shift, with most of these (32, 74%) being upregulated at 37°C (Figure 1; Materials and Methods). The vast majority of the genes, 94%, were transcribed only from the strand containing the ORF, thus allowing quantification and comparison of the gene expression with strand-insensitive sequencing.

At the two temperatures used in our transcriptome analysis, we observed few significant changes in the expression of cold-response (*capB* and PA14_05960) or heat-shock genes (*htpG*), and did not find evidence for extensive cold- or heat- shock response that is indicative of thermal stress. This suggests that *P. aeruginosa* has evolved to thrive under a range of conditions that may include a variety of environmental temperatures without the need to activate protective survival responses.

### The type III secretion system is preferentially expressed at 37°C

**Figure 2:**
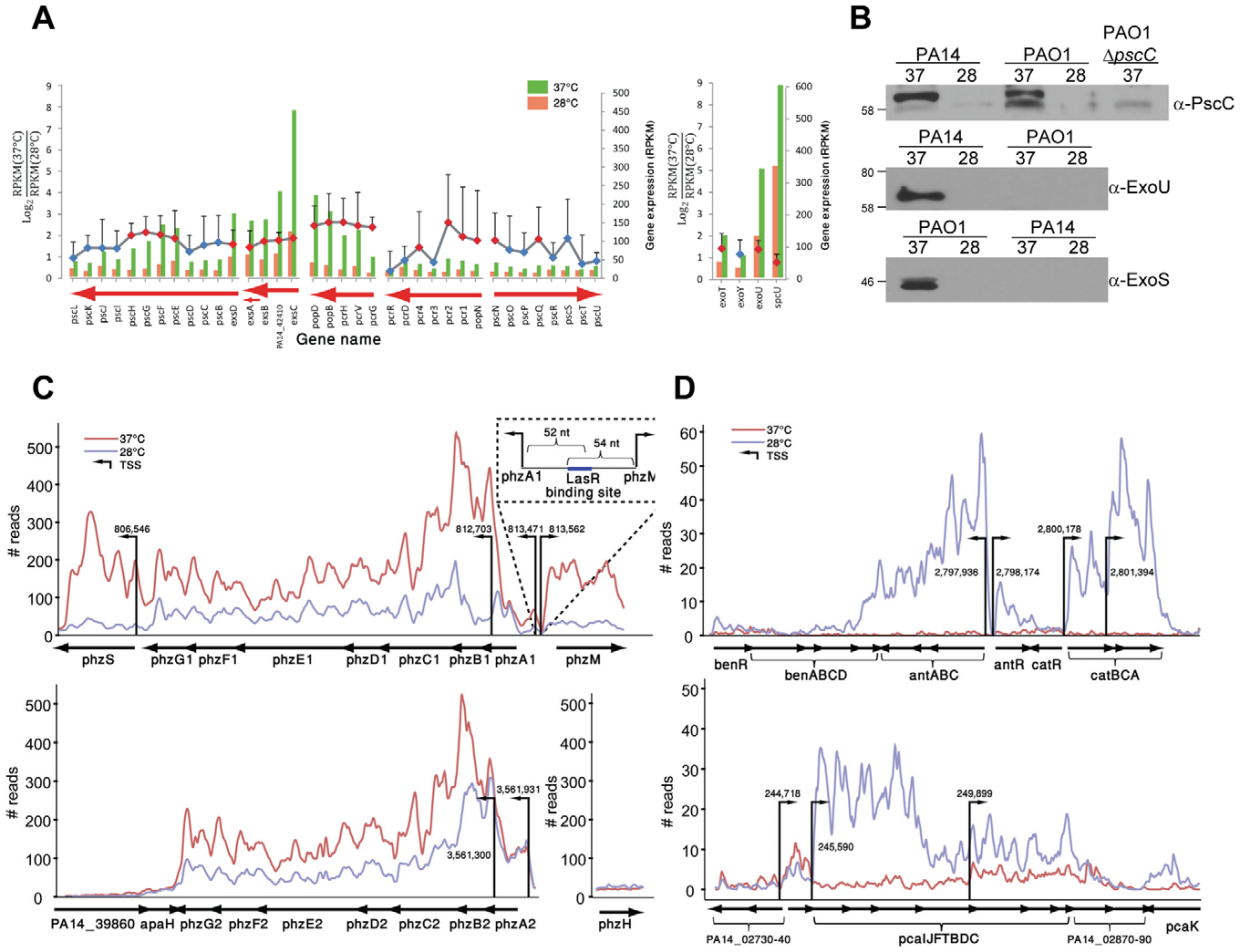
Temperature-regulated expression of selected virulence factors. (A) The T3SS of *P. aeruginosa* is induced at 37°C. Transcript levels of the genes encoding the T3SS components; its regulators; secreted effectors ExoT, Y, U; and the chaperone SpcU. Normalized gene expression [78] (Materials and Methods) for each gene in the two growth temperatures is represented by bars for each gene (green and red, 37°C and 28°C, respectively). The log_2_-ratio for 37°C and 28°C expression is represented by a diamond for each gene [red diamonds represent a statistically significant difference (χ^2^, p<0.05) in expression for two replicates while genes marked with blue diamonds did not pass the significance threshold]. Genes connected by a line between their representing diamonds are part of the same transcriptional unit (TU). Error bars represent a single standard deviation from the mean. Horizontal red arrows represent TUs and their strand-orientation (right and left, forward and reverse strand, respectively). (B) Analyses of expression of the secretin and exoproteins of the T3SS in strains PA14 and PAO1. Cellular (top panel) and secreted (middle and lower panel) proteins from cultures growing at 37 or 28°C (labeled 37 or 28 respectively) were separated by SDS-PAGE followed by Western immunoblotting using antibodies against PscC (α-PscC), ExoU (α-ExoU), or ExoS (α-ExoS). The migration of molecular protein standards are indicated in kilodaltons. The common band on all blots probed with the α-PscC antibody represents a cross-reactive protein. (C) Expression of genes for the phenazine biosynthesis operons. The normalized number of sequencing reads mapped to the genes (black arrows) in the phenazine biosynthesis operons is shown in 37°C and 28°C (red and blue lines respectively). The TSSs that were detected in the operons are shown as black vertical lines (arrowheads representing the strand of the transcription, right and left, forward and reverse, respectively; numbers represent the genomic position of the TSS). A palindromic LasR binding site was identified 52 nt upstream to the TSS of the *phzA1-G1* operon and 54 nt downstream of the TSS of *phzM*. (D) Expression of genes for enzymes in pathways that contribute precursors to the biosynthesis of phenazines.

The mRNA levels for the majority of the T3SS components (regulators, secretion machinery and effectors) were higher at 37°C (Figure 2A) suggesting that this important *P. aeruginosa* virulence mechanism is subject to temperature regulation. Since the RNA-seq was carried out in calcium replete media, which is not optimal for the expression of the T3SS genes, we analyzed the production of the secretin component PscC by Western blots in two different strains, PA14 and PAO1, grown on calcium-depleted media, which promotes T3SS expression (Figure 2B). In both strains we observed immunoreactive PscC at 37°C but not at 28°C. Similarly, we observed temperature-regulated production of species-specific effectors of the T3SS, ExoS and ExoU in PAO1 and PA14, respectively, where they were detected only at 37°C (Figure 2B), confirming that the temperature regulation is not a PA14-specific trait.

The observed regulatory effect of temperature on the T3SS suggests that this virulence mechanism should play an important role in *P. aeruginosa* infections of warm-blooded mammals. The importance of the T3SS for acute human nosocomial infections has been demonstrated and T3SS-deficient *P. aeruginosa* strains are attenuated in murine models of pneumonia, bacteremia, keratitis and burn wound infections [31]. However, the T3SS has been shown to contribute to virulence in models where the hosts were infected at their optimal growth temperatures, such as *Drosophila melanogaster* [32], *Acanthamoeba castellani* [33], *Galleria mellonella* [34] and more recently, *Danio rerio* (zebrafish) embryos [35], [36] although this virulence mechanism appears non-essential for the infection of adult gnotobiotic fish [37]. *P. aeruginosa* can also infect *Caenorhabditis elegans*, although its T3SS is expressed but not required for virulence [38]. Since the T3SS contributes to the virulence of *P. aeruginosa* even at reduced temperatures, it is conceivable that the environment of specific hosts provides additional signals that overcome those provided by the temperature or other environmental factors. Previous work has shown that contact between *P. aeruginosa* and mammalian cells can trigger the expression of T3SS even in the presence of calcium concentrations that *in vitro* inhibit the expression of these genes [39].

### Effect of growth temperature on the expression of genes encoding phenazine biosynthesis enzymes

The second group of genes showing a significant increase in mRNA levels encodes enzymes responsible for the biosynthesis of phenazines, which are well characterized virulence factors of *P. aeruginosa* [40]. Phenazines are nitrogen-containing heterocyclic secondary metabolites that serve as signaling molecules influencing gene expression during environmental adaptations including biofilm formation [41], [42]. Phenazines are capable of producing reactive oxygen species toxic to eukaryotic cells and other bacteria [43], [44]. These molecules are also involved in electron shuttling to alternate terminal acceptors particularly during anaerobic growth [45]. Moreover, the production of phenazines in other *Pseudomonas* species is temperature regulated [46]. In *P. aeruginosa*, two unlinked gene clusters, *phzA1-G1* and *phzA2-G2* encode enzymes catalyzing the synthesis of the core molecule phenazine-1-carboxylic acid (PCA) [47]. Although levels of transcripts from both of the *phz* clusters were elevated at 37°C, the temperature effect was more pronounced for *phzA1-phzG1* (Figure 2C). Two additional genes located adjacent to the *phzA1-G1* operon encode enzymes involved in the modification of PCA: PhzM (a methyltransferase) and PhzS (a monooxygenase). These two enzymes modify PCA to give pyocyanin, while PhzS alone can convert PCA to 1-hydroxy-phenazine [48]. The levels of *phzM* and *phzS* mRNAs were also increased at 37°C. Finally, the *P. aeruginosa* chromosome contains the *phzH* gene at an unlinked site; it encodes the enzyme for the conversion of PCA to phenazine-1-carboxamide. However, we did not detect significant expression of this gene at either temperature, and, therefore, it is unlikely that this modification step takes place in *P. aeruginosa* under the conditions tested.

Phenazine is synthesized by a pathway utilizing metabolites that are also precursors for aromatic amino acids, TCA cycle intermediates and other bioactive molecules [49]. We therefore examined the levels of transcripts for enzymes of the entire pathway and its branches, starting from condensation of erythrose-4-phosphate and phosphoenol pyruvate to various end products (Figure S1). Genes found in several operons encoding enzymes of these pathways were significantly more expressed (ranging from 6.6- to 35-fold) at 28°C than 37°C. Specifically, *antABC*, the genes for enzymes catalyzing the synthesis of catechol from anthranilic acid, and genes encoding CatABC and PcaDIJF, responsible for the conversion of catechol to the TCA cycle intermediates succinate and acetyl-CoA, were preferentially expressed at 28°C when compared to 37°C (Figure 2D). We also detected temperature regulation of the *antR* gene encoding the transcriptional activator of the *ant* operon, which was not the case for mRNA levels of *catR*, the regulatory gene of the *cat* operon. Although numerous regulatory inputs are involved in regulating these genes at the transcriptional and post-transcriptional levels [50], appears that the conversion of anthranilate to TCA cycle intermediates may occur less effectively at 37°C than at 28°C, making more precursors available for other biosynthetic pathways.

**Figure 3:**
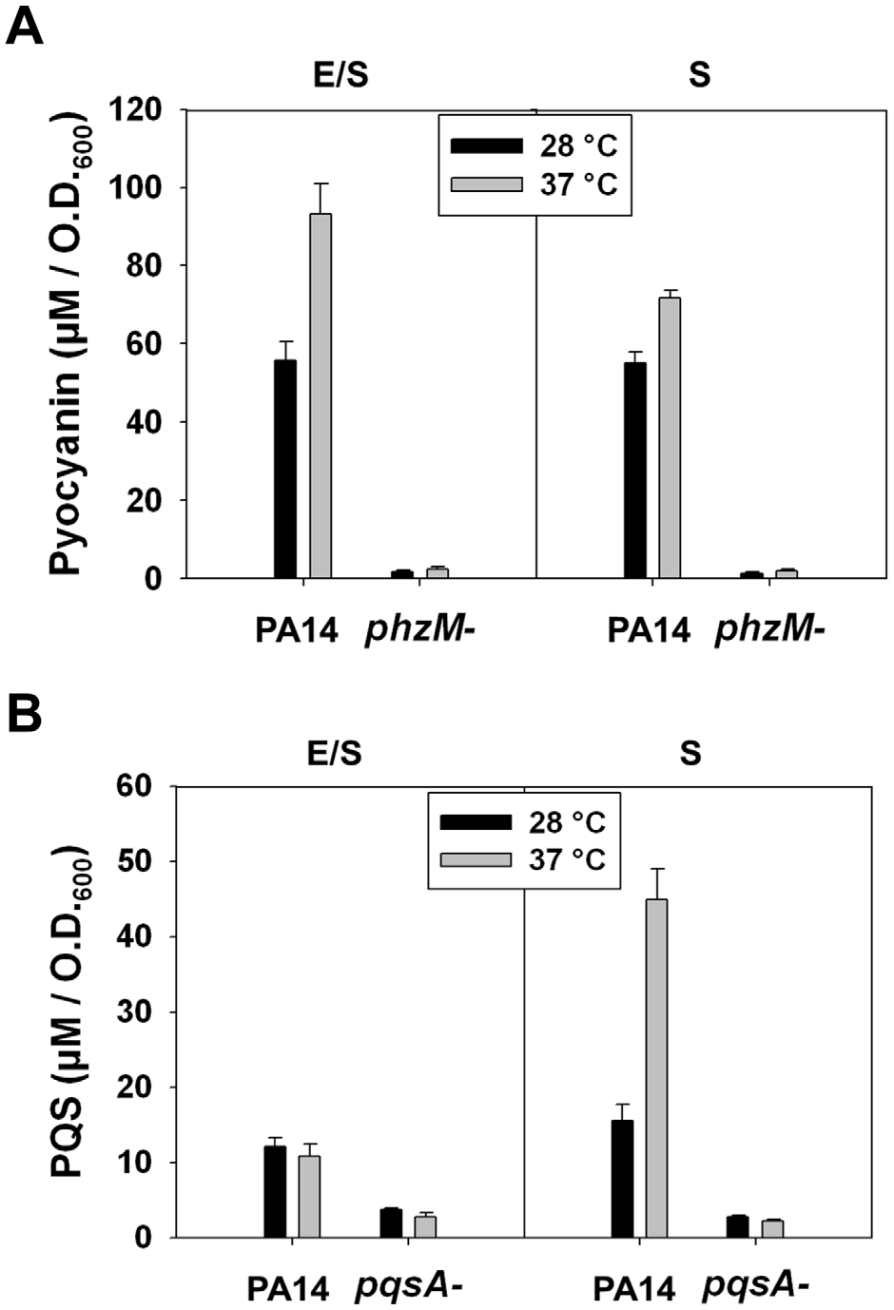
Effect of temperature on production of pyocyanin and PQS. The growth kinetics of cultures of *P. aeruginosa* PA14 were monitored at O.D._600_. The levels of pyocyanin (A) and PQS (B) were determined at 28°C and 37°C at the time of transition from exponential to stationary phase (E/S) and at stationary phase (S). Similarly grown cultures of *P. aeruginosa* Δ*phzM* and Δ*pqsA* were used as negative controls for pyocyanin and PQS production, respectively.

Our results therefore show that at 37°C there should be a significant increase in the levels of two metabolic intermediates affecting the synthesis of important signaling and virulence-enhancing molecules (Figure S1). An increase in the concentration of chorismic acid would be the consequence of higher levels of PhzC at 37°C and constitutive, temperature-independent transcription of genes encoding enzymes of the shikimate pathway (AroF1BQ1EKAC). Chorismic acid can be also converted to anthranilic acid, which would accumulate at 37°C, in part because of a decrease in its flow through the breakdown pathway to the TCA cycle intermediates succinate and acetyl-CoA, resulting from lower levels the enzymes (CatABC, PcaDIJF). An important consequence of this redistribution of various metabolic intermediates at 37°C is the diversion of chorismic acid towards the biosynthesis of phenazine. Anthranilic acid is also a precursor for the biosynthesis of the quorum sensing molecules alkyl-4 quinolones (AQs). Therefore, the reduction of the levels of the *ant*, *cat* and *pca* transcripts at 37°C (and by inference, levels of corresponding catabolic enzymes) could lead to an increase of the production of 2-heptyl-4-quinolone (HHQ) and 2-heptyl-3-hydroxy-4-quinolone (PQS), two important regulators of *P. aeruginosa* cell-to-cell communication and virulence gene expression [51], [52]. We tested these predictions directly by comparing the levels of pyocyanin and PQS in *P. aeruginosa* supernatants of cultures grown at 28°C and 37°C (Figure 3). We found increased concentrations of both of these molecules at 37°C, although the effect on PQS production was much more pronounced when cultures reached the stationary phase of growth. Moreover, the pyochelin siderophore pathway also benefits from the altered levels of the various precursors of the aromatic amino acid pathway, since increased levels of chorismic acid as the consequence of a decrease in its flow towards the TCA cycle makes more of it available for the synthesis of pyochelin, although mRNA concentrations encoding the enzymes of this pathway were not affected by temperature.

The temperature-dependent redirection of transcripts encoding the components of the aromatic amino acid biosynthetic machinery, towards the synthesis of phenazines and signaling molecules such as AQs, provides new insight into the role of environmental modulation of global survival strategies of *P. aeruginosa*. Although our study focused on the influence of temperature on transcript levels, the precise molecular mechanisms that accomplish thermoregulation are unclear. However, the results of this RNA-seq study provide a basis for the design of rational genetic and biochemical experiments to probe the molecular details of signal transduction, gene expression and protein function at different temperatures that are undoubtedly coordinated with other physical or chemical environmental inputs.

### The transcriptome structure of *P. aeruginosa* PA14

Understanding the transcriptome structure and operon organization in bacteria is essential for understanding bacterial RNA-based regulation [19]. To produce a detailed transcriptome structure map of *P. aeruginosa*, we used the mapping of the RNA-seq reads, which provides transcript coverage in a non-strand-specific manner. To complement this approach, we also used a strand-specific 5′-end sequencing method, which identifies active transcription start sites (TSS) at a single nucleotide resolution across the entire genome [15], [17]. The combination of the two sequencing methods provided a comprehensive view of the *P. aeruginosa* PA14 transcriptome in an unbiased manner.

We were able to map transcription start sites (TSSs) for 2,117 transcriptional units (TU), spanning 3,325 protein-coding genes (55% of all protein coding genes, Table S3). A total of 1,854 genes (56%) were found to be included in polycistronic TUs (Figure S2A). Over 61% of the multi-gene operons are bicistronic, and only 19% contain 4 or more genes. This general operon organization is highly similar to that found in other bacteria. For example, in the Gram-positive *Listeria monocytogenes*, in which the operon structure was determined by hybridization of RNA to tiling-arrays, it was found that 60% of the genes were transcribed in multi-gene operon structures, with about half of the multi-gene TUs transcribed as bicistronic mRNAs [53]. In *Geobacter sulfurreducens*, polycystronic mRNAs accounted for approximately 50% of all transcripts [54] and a recent determination of *Escherichia coli* K12 found that 35% of the TUs are polycistronic [55]. Since these organisms vary greatly in genome size, growth conditions and physiology, there appears to be a general design principle for bacterial operon organization, which has no correlation with the genome coding capacity. We note that TU structures can be flexible and may change when conditions change [56], [57]; however, since in this study we tested only two growth conditions, a global analysis of alternative TU structures could not have been performed.

### Definition of transcriptional units and the analysis of 5′ untranslated regions (5′ UTRs)

The capacity of 5′ UTRs to regulate transcriptional and post-transcriptional processes in *cis* has been demonstrated in numerous organisms [58]. Until now, the mapping of 5′ UTRs has not been performed systematically on *P. aeruginosa*, thus limiting the discovery of new *cis*-regulatory elements. Since our TSS mapping defines the 5′ ends of TUs, it also reveals the 5′ UTRs of the immediate downstream genes. Inspection of the 5′ UTRs for the 2,117 TUs we defined showed a median 5′ UTR length of 47 nt (Figure S2B), similar to the 5′ UTR lengths reported in *E. coli* (20–40 nt) [59] and *Synechococcus elongatus* (30 nt) [18]. Most of the 5′ UTRs (77%) were shorter than 100 nt, with 60 genes completely lacking 5′ UTRs. Interestingly, 115 5′ UTRs were longer than 200 nt, which suggests that a significant fraction of these leader regions may function as *cis-*regulatory RNA elements. We scanned the sequences of all 5′ UTRs longer than 100 nt using RFAM, and found that 13 of those contain known riboswitches and RNA-leaders (Table S3). Since most of the well-characterized riboswitches models derive from highly divergent bacteria, such as *Bacillus subtilis*, the presence of these long 5′ UTRs suggests that many post-transcriptional regulatory sequences in the *P. aeruginosa* are yet to be discovered.

The availability of genome-wide TSS maps can allow the correction of false computational gene annotations [19]. Indeed, we were able to correct the annotation of 46 genes (Table S3).

### LasR-regulated genes

**Figure 4:**
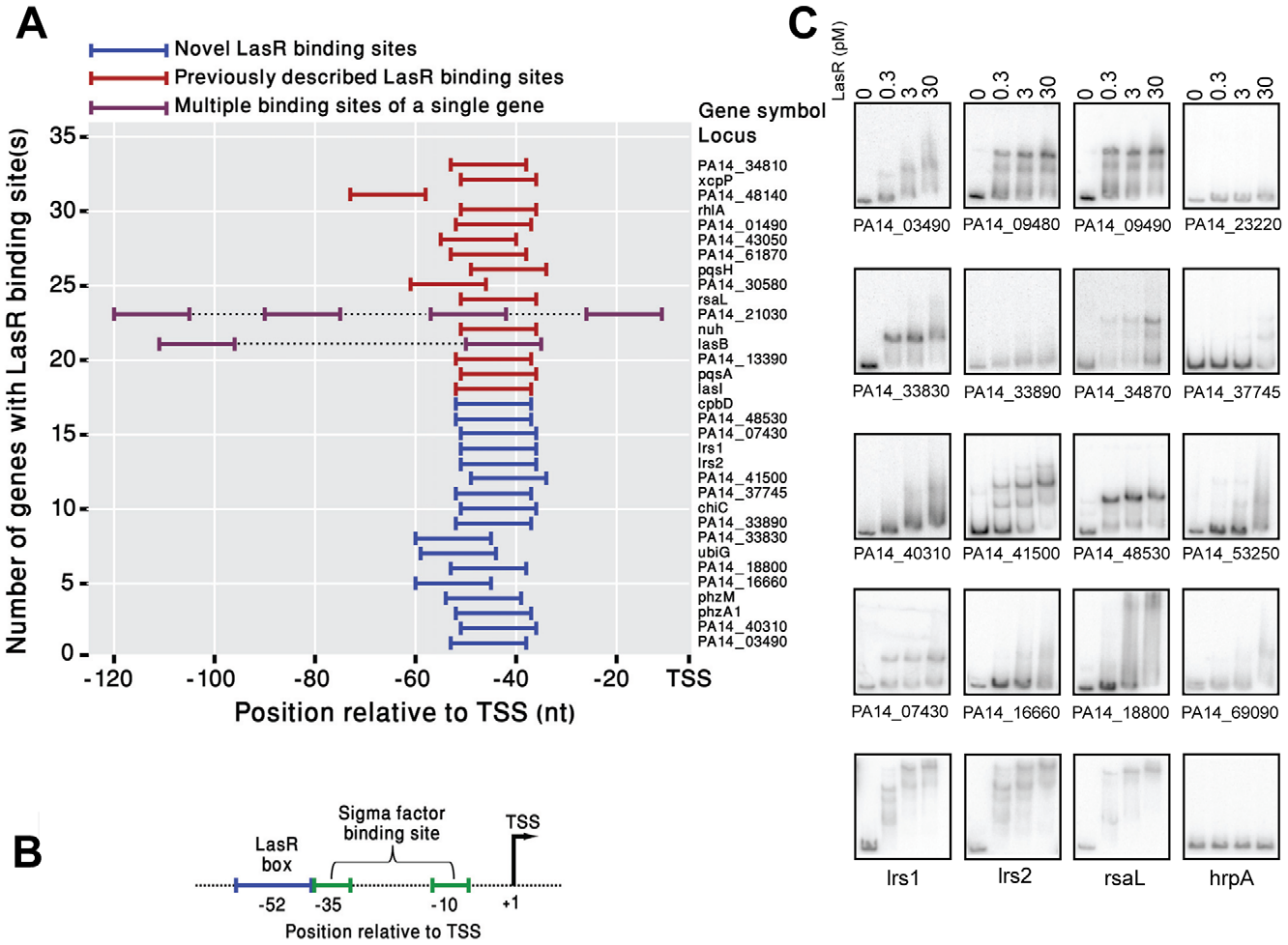
Identification of new LasR binding sites. (A) Location of LasR boxes of genes relative to the TSSs. Most LasR binding sites (LasR box) are localized at 51–52 nt upstream to the TSS of the genes. The binding site is shown as a line upstream to the TSS (blue, red, and purple; novel binding sites, previously known binding sites, and genes with multiple binding sites, respectively). (B) Positioning of the LasR box (blue line) is immediately upstream to the sigma factor binding site (green line). Black arrow represents the TSS. (C) EMSA of DNA fragments from predicted LasR-regulated genes. The concentrations of purified LasR used in each assay are shown above the reactions. DNA fragments from the promoter region of the *rsaL* and an internal fragment from the *hrpA* gene were used as positive and negative controls, respectively.

Since gene annotation in *P. aeruginosa* is largely limited to the description of ORFs, a genome-wide characterization of promoter regions and binding sites of regulators of transcription is often difficult. The availability of a comprehensive list of TSSs in *P. aeruginosa* now allows an accurate search for promoter and regulatory regions for specific genes or operons. To demonstrate the utility of this approach, we selected LasR, the transcriptional regulator of quorum sensing and virulence genes. LasR independently regulates the expression of a number of genes. It is positioned at the top of a regulatory network that includes additional quorum sensing systems such as Rhl, PQS and Qsc and, therefore, its regulon is extensive [60]–[62]. The number of genes directly or indirectly controlled by LasR was shown to include over 300 genes or about 6% of the genome [63], [64]. A recent chromatin immunoprecipitation coupled to microarray hybridization study (ChIP-chip) [65] has characterized 35 LasR binding sites in the *P. aeruginosa* PAO1 genome, but the lack of TSS information prevented accurate localization of the LasR sites relative to the transcript. By analyzing the TSSs of genes marked as LasR-bound by the ChIP-chip study, we were able to establish that this transcription factor has a preferential positioning of 51–52 bases upstream to the start of transcription (Figure 4A). Based on this analysis, we computationally identified 17 new, previously unrecognized putative LasR binding sites on the *P. aeruginosa* PA14 genome (Table S4; Materials and Methods). An additional 20 sites, previously shown to be bound by LasR in strain PAO1 [7], [64], [65] were also identified in the PA14 strain. The vast majority of the sites recognized by LasR were immediately adjacent to the −35 element of the promoter, making it likely that it contacts RNA polymerase during transcription initiation (Figure 4B). Two genes, PA14_21030 (ATP-dependent Clp protease subunit) and PA14_16250 (*lasB*, previously identified by Gilbert et al [65]) contained multiple LasR binding sites at their promoters (Figure 4A).

To confirm that the new LasR binding sites identified by our computational methods represent direct targets of regulation by this transcription factor, we analyzed these promoters for direct binding by LasR using electrophoretic mobility shift assays (EMSAs) and for LasR-dependent expression using *lacZ* transcriptional fusions. When purified LasR was used to analyze DNA-protein interactions, we detected binding of this protein to all but one fragment (Figure 4C). Based on the low concentrations of LasR (0.3 pM) that were required to give a complete or near-compete shift, relatively high affinity binding was detected for at least five DNA fragments corresponding to the promoter regions of PA14_09480, PA14_09490, PA14_33830, and PA14_48530. For the rest of the DNA fragments, the extent of binding of LasR varied and in a number of cases, only a weak shift was observed even at the highest concentrations (30 pM) tested. No binding of LasR to the promoter region of PA14_23220 was detected.

To determine whether the newly identified LasR-dependent binding sites indeed respond to LasR *in vivo*, we have cloned the same fragments used in the EMSAs into a transcriptional *lacZ* reporter plasmid and incorporated the gene fusion into the chromosome of wild-type *P. aeruginosa* PA14 and its isogenic *lasR* mutant. LasR-dependent *lacZ* expression was assessed at two time points corresponding to early stationary phase (8 hr growth) and late stationary phase (12 hr growth). In general, the levels of expression for most of the fusions, and correspondingly LasR dependence, were higher at later stages of growth (Figure S3). A number of fusions were poorly expressed in *P. aeruginosa*, and because of this, no LasR-dependent regulation was observed for the fusions transcribed from the promoters of PA14_03490, PA14_23220, PA14_18800, and PA14_33890. This lack of expression is consistent with the transcriptome data (RPKM values of 30 or less for these genes) suggesting they are not expressed under these conditions. Interestingly, we observed LasR binding to three of these promoters (PA14_03490, PA14_18800, and somewhat weakly, PA14_33890) in our *in vitro* assay. Two conclusions could be drawn from these results. First, the predicted LasR binding sites adjacent to promoter regions do not necessarily indicate that a particular gene is expressed under all conditions. For example, PA14_03490, PA14_18800, and PA14_33890 could be expressed in environments that are different from these used in our work (LB, aerobic and at 37°C). Moreover, full transcriptional activation of certain LasR-regulated genes could require the input of additional regulatory factors. This regulatory complexity of quorum sensing would permit a more fine-tuned response of groups of genes to specific environmental inputs.

### Regulation of phenazine biosynthetic operons

The *P. aeruginosa* PA14 genome contains two temperature regulated gene clusters, encoding the enzymes for the synthesis of phenazine-1 carboxylic acid (*phzS*, *phzA1-G1*, *phzM*, and *phzA2-G2*; Figure 2C). Regulation of the *phzA1-G1* operon and *phzM* by acyl-homoserine-lactones (HCLs) has been shown previously using DNA microarrays [63], [64]. Using the precisely-mapped TSSs, we independently identified in the *phzA1*-*phzM* intergenic region a conserved palindromic LasR consensus binding sequence C TACCAGATCTTGTAG, with the C positioned 52 nt from the site and on the opposite strand, the sequence C TACAAGATCTGGTAG with the C 54 nt from the TSS of *phzM* (Figure 2C, 5A). Since the consensus LasR binding site is palindromic, the binding of this transcription factor to a single site in an intergenic region could allow activation of the two divergent promoters, a rather uncommon arrangement of regulatory sequences bound by positive transcription regulators in bacteria. Moreover, since the LasR binding site resembles the predicted binding site for a second quorum sensing regulator RhlR [61], [63], and LasR regulates the expression of *rhlR*, it is conceivable that the same palindromic double stranded DNA sequence serves as a binding site for either LasR or RhlR, or both for these two transcriptional units.

**Figure 5:**
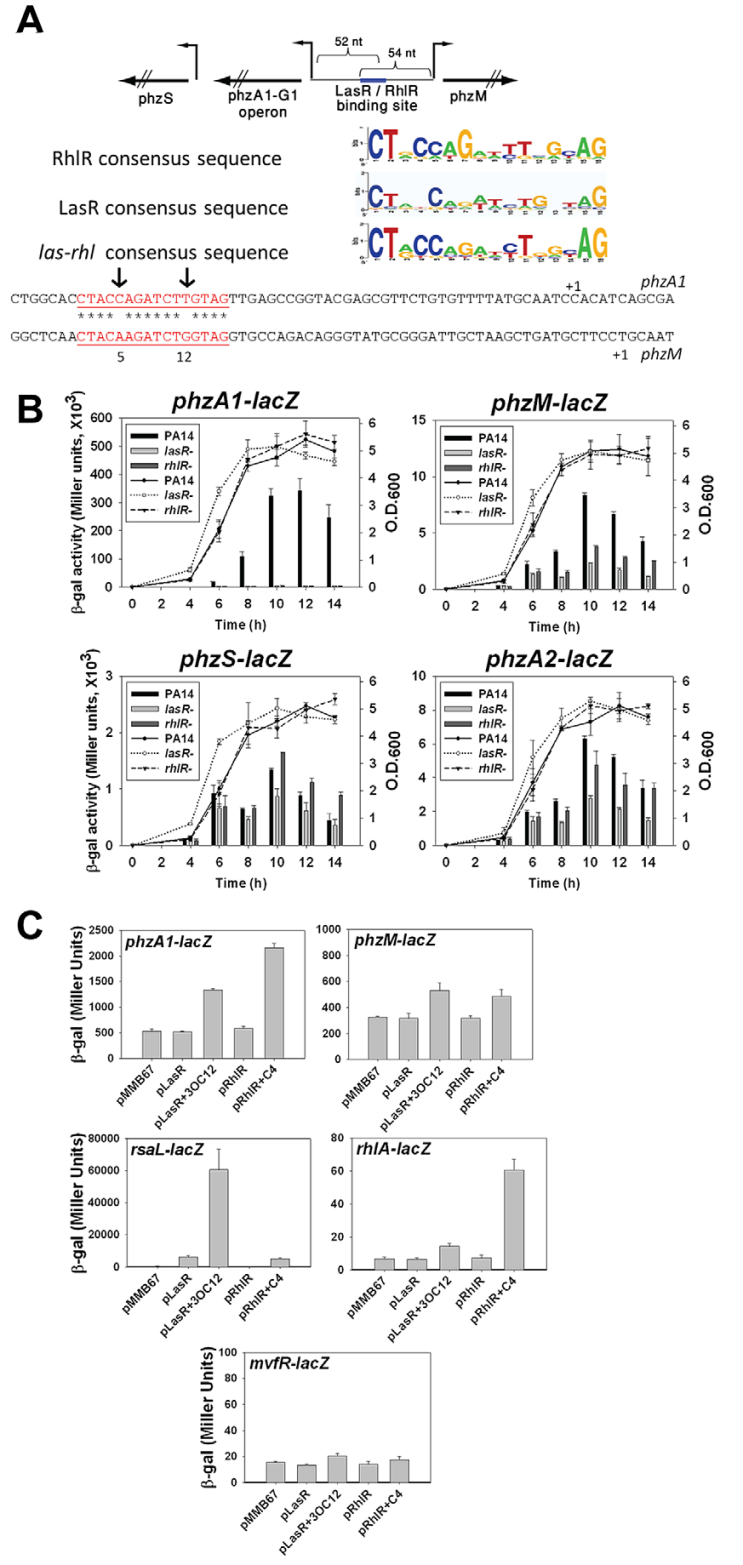
Role of LasR and RhlR in regulating the biosynthesis of pyocyanin. (A) The location of the LasR and/or RhlR binding sites are presented on top. Binding sequences are highlighted in red, asterisks in the binding sequences represent nucleotide identity between the forward and reverse sequences of the palindromic binding site; non-identical positions are marked with arrows. Also shown are the consensus sequences recognized by LasR, RhlR and the sequence of the LasR-RhlR box taken from previous studies [63], [65], [90]. (B) Growth (lines) and activities of the *lacZ* reporter (bars) fused to various promoters fragments of the *phz* operon in wild-type *P. aeruginosa* PA14, Δ*lasR* and Δ*rhlR* strains. (C) Promoter activity in *E. coli*; (i) a fusion of a promoter fragment and *lacZ* transcriptional reporter (indicated in the upper left corner of the panel) and (ii) plasmid expressing either *lasR* (pJTT201, indicated as pLasR) or *rhlR* (pJTT202, indicated as pRhlR), or the empty vector pMMB67 [73]. The acyl-homoserine lactones 3OC_12_-HSL (2 µM) and C_4_-HSL (10 µM) were added as indicated. The *rsaL* promoter previously shown to directly bind LasR was used as a positive control while a fragment containing the *mvfR* gene's promoter served as the negative control.

Promoter-*lacZ* fusion experiments in wild-type, *lasR*, and *rhlR* mutants of *P. aeruginosa* showed strong LasR- and RhlR-dependent expression of the *lacZ* reporter fused to the *phzA1* promoter fragment (Figure 5B). Similarly, the *phzM* promoter directed transcription in a LasR- and RhlR-dependent manner, although the fold decrease of transcription of the fusion in *lasR* and *rhlR* mutants was not as large as seen for the *phzA1*-*lacZ* fusions, owing to its high level of expression in wild-type *P. aeruginosa*. There was only modest regulation by LasR or RhlR for the *phzS* and *phzA2* fusions (Figure 5B).

Because LasR controls the expression of *rhlR*, it was impossible to differentiate which of the two quorum sensing transcription factors regulated the expression of *phzA1* or *phzM*. We therefore engineered *phzA1-lacZ* and *phzM-lacZ* promoter fusion plasmids and the expression of the reporter gene was measured in response to N-(3-oxododecanoyl) homoserine lactone (3O-C12-HSL) or N-butyryl homoserine lactone (C4-HSL), in *E. coli*, co-expressing LasR or RhlR on an inducible plasmid. DNA fragments containing the promoter regions of *rsaL* and *rhlA* were used as controls for specificity of expression of activation of the reporter constructs by LasR and RhlR, in response to 3O-C12-HSL and C4-HSL, respectively (Figure 5C).

An approximately 3.7-fold and 6-fold induction of *phzA1*-*lacZ* was seen in *E. coli* expressing either LasR or RhlR, in response to their cognate autoinducers. The *phzM-lacZ* fusion showed a significant, albeit modest, effect of the homoserine lactone autoinducers and LasR, RhlR (1.6-and 1.3-fold, respectively). The results from the expression of the *phzA1-lacZ* and *phzM-lacZ* fusions in *P. aeruginosa* and heterologous expression in *E. coli* indicate that binding of LasR and RhlR to the same site, in the context of the respective promoters, leads to the enhanced expression of *phzA1* over *phzM* in overall magnitude and dependence on these two quorum sensing regulators.

There are several explanations for these observations. First, the two overlapping regulatory sequences found on the opposite DNA strands are not functionally identical although the differences are rather minor (Figure 5A). There are two differences in the regulatory sequences adjacent to each promoter (T/A at position 5 and C/G at position 12). These could account for strong preference for LasR and RhlR activation towards the transcription of *phzA1*. Moreover, the predicted promoters (−10 and −35 regions) are not identical and these could also provide additional context for the extent and magnitude of LasR and RhlR activation. Finally, the distances of the LasR element from the TSSs of *phzA1* and *phzM* are different, although well within those seen for other LasR activated genes, and this could also contribute to the overall transcriptional level and extent of influence of quorum sensing regulators and autoinducers. Conceivably, all of these factors could incrementally contribute to regulated expression.

### Detection of expressed non-coding RNAs

**Figure 6:**
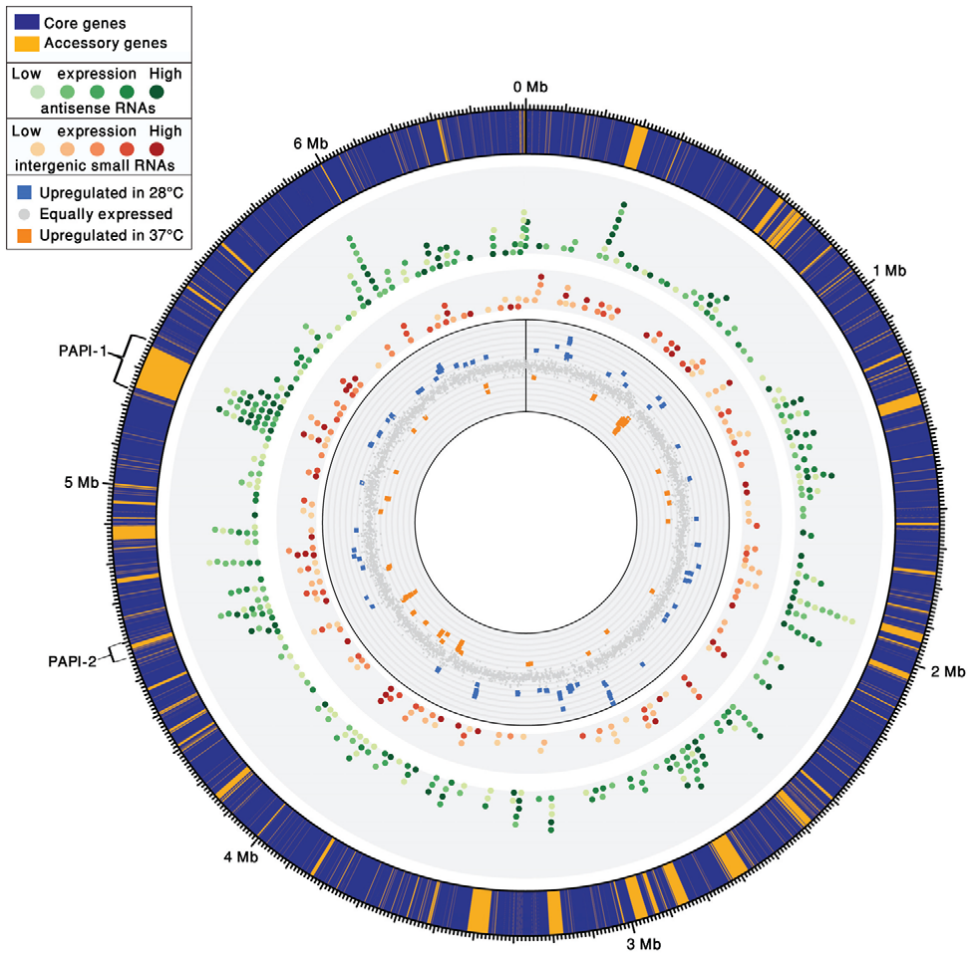
Distribution of temperature regulated genes, and segments transcribing antisense RNAs and intergenic small RNAs in the genome of *P. aeruginosa* PA14. The outer circle represents the core (blue) and accessory genes (yellow) as defined previously [9]. The innermost two circles represent genes that were differentially expressed at 28°C and 37°C (blue and orange, upregulated in 28°C and 37°C, respectively); the middle two circles show the expression of sRNAs from intergenic regions (red dots) and asRNAs (green dots). For each category, relative ranking of expression was grouped into five color-coded bins (darker colors represent higher expression).

Non-coding RNAs (ncRNAs) are now appreciated as important regulators of diverse processes in bacteria. Although *P. aeruginosa* has been studied extensively, only 38 intergenic ncRNAs (sRNAs) have been identified in its genome so far [66]. By combining our TSS mapping and the whole-transcriptome data, we determined 223 events of intergenic transcription based on the current genome annotation (Figure 6). Of these, 23 represent protein-coding genes that were annotated in other genomes but so far escaped detection in PA14 (Table S5; Materials and Methods), and 35 additional transcripts that might represent non-conserved protein coding genes (Table S6). The sequences of the remaining 165 transcripts lack an ORF and therefore most likely represent non-coding transcripts (Table S6). Most of the previously described sRNAs (31/38, 86%) were detected in our sRNA set, and we could define the exact TSS for 26 of them. Most of the sRNAs we identified (69.5%) are conserved only within other *P. aeruginosa* strains, and only a few (10.5%) are conserved outside of the genus *Pseudomonas* (Table S6).

In addition, we identified 384 genes with overlapping transcription on the reverse strand, representing *cis*-encoded antisense RNA (asRNA) transcription, Such asRNAs are known to be abundant regulators in many prokaryotes [15]–[19], [53], [54] but until now they have not determined globally in *P. aeruginosa*.

We next examined the distribution of the DNA segments that are transcribed into asRNAs and sRNAs in the genome of *P. aeruginosa* PA14. Specifically, we assessed whether they are part of the flexible or core genome, as determined previously [9] (Figure 6). We noted an over 2-fold enrichment in the location of sequences specifying both sRNAs (Fisher's exact test p = 0.003) and antisense RNAs (Fisher's exact test p = 8.83×10^−18^) in regions that have been previously designated as regions of genome plasticity (i.e. the flexible genome); sRNAs and asRNAs were found less frequently in the conserved core genome. For example, the annotated 115 kb pathogenicity island PAPI-1 carries only two genes with predicted function in transcriptional regulation, RL0037 (a RcsB ortholog) and a predicted transcription regulator RL0012 [67]. Strikingly, in addition to the 175 annotated genes in PAPI-1, we were able to detect additional 46 ncRNAs (10 sRNAs and 36 asRNAs) representing 21% of the genes in the island; this number of asRNAs in PAPI-1 is enriched by over 5-fold compared to the core genome. Since the flexible genome represents, in most instances, horizontally-acquired genes, it is likely that these segments specify regulatory elements controlling the expression of co-acquired target genes. However, it could be that genes expressed from other genomic islands or from the core genome could be subject to regulation by the *trans*-acting sRNAs at a post-transcriptional level [68]. These findings raise the possibility that the ability to acquire ncRNA regulators via horizontal gene transfer contributes significantly to bacterial evolution, where new phenotypes emerge by post-transcriptional activation or repression of genes already present in the recipient cells. An alternative explanation is that horizontally acquired sequences in the flexible genome were newly introduced to the genome, and due to lack of evolutionary time to ameliorate their coding- and non-coding sequences, the acquired sequences contain random promoter sequences that are recognized by *Pseudomonas* transcriptional machinery.

**Figure 7:**
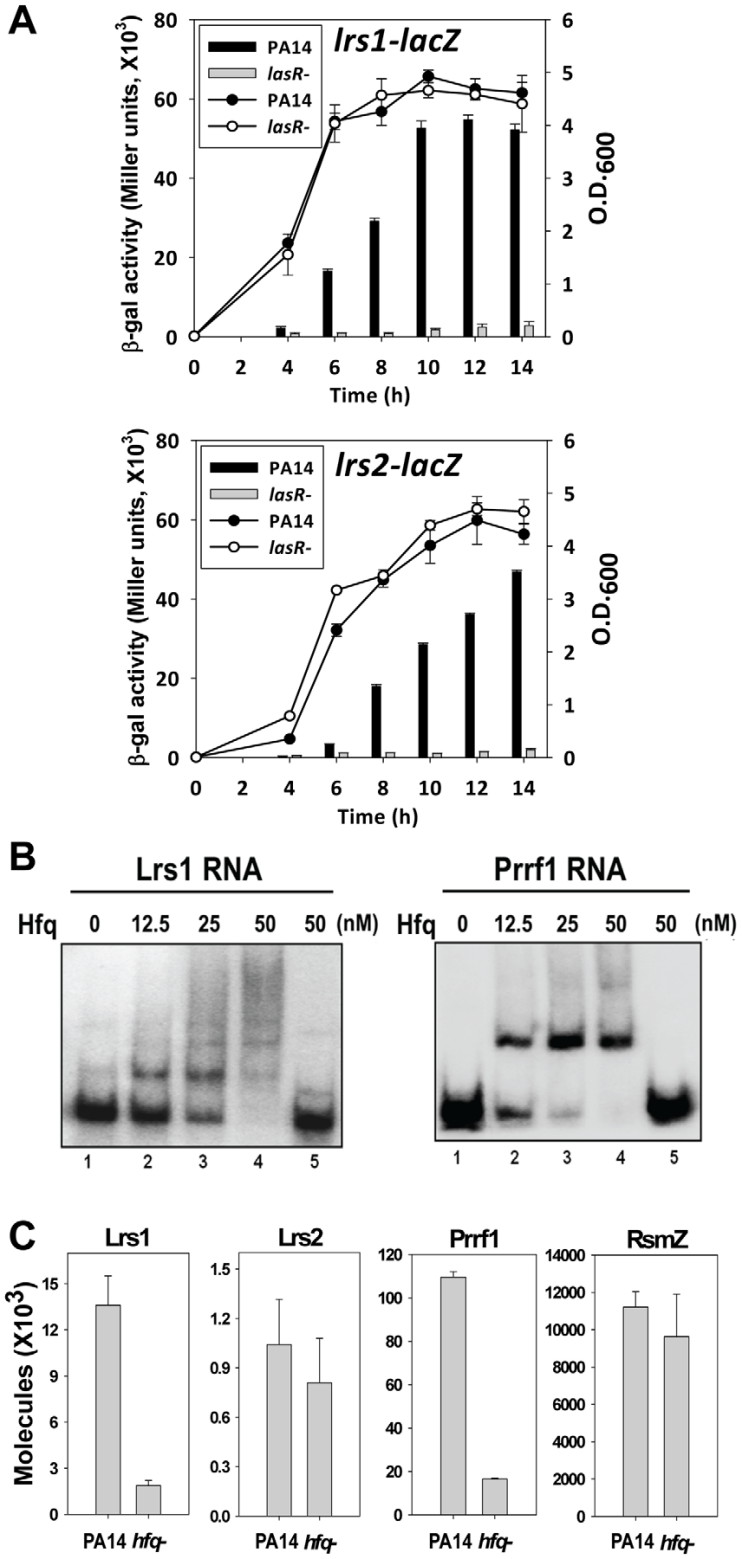
Characterization of the LasR-regulated small RNAs Lrs1 and Lrs2. (A) Growth (lines) and expression of the *lrs1* or *lrs2* reporter constructs (bars) in wild-type or Δ*lasR P. aeruginosa* PA14 are shown. (B) Role of Hfq in controlling the levels of Lrs1 and Lrs2 sRNAs. Gel shift analyses of sRNAs interaction with increasing amounts of purified Hfq are shown. Lane 5 in both panels contains, in addition to labeled probe, a 100-fold excess of unlabeled Lrs1 or Prrf1 RNA. (C) Lower panels show RT-PCR analyses of Lrs1 and Lrs2 expression in *P. aeruginosa* wild-type and Δ*hfq*. Prrf1 and RsmZ were used as positive and negative controls, respectively, for sRNAs interacting and not interacting with Hfq.

Interestingly, two of the predicted sRNAs (Lrs1 and Lrs2) were identified as having a conserved binding site with similar binding affinity for LasR (Figure 4A, 4C). Monitoring the expression of *lrs1* and *lrs2* with transcriptional *lacZ* fusions showed LasR dependence (Figure 7A) throughout the entire growth phase of the cultures, although there was a slight increase in the expression of the *lrs1-lacZ* fusion and *lrs1* transcript levels (Figures S3 and S4B) when cultures reached a late stationary phase of growth. A Northern blot with a probe designed for *lrs1* with RNA extracted from wild-type PA14, a Δ*lasR* mutant, and a Δ*lasR* strain complemented with LasR on a plasmid indicated that *lrs1* expression is indeed dependent on LasR (Figure S4B). We also note that during the late stationary phase Lrs1 can be detected even in a *lasR* mutant, suggesting that its regulation may be directed by another transcription factor expressed or activated at this late growth phase. We further demonstrated using EMSA that the concentrations of Lrs1 (but not Lrs2) in *P. aeruginosa* were dependent on the RNA chaperone Hfq through a direct interaction (Figure 7B). We also observed that Lrs1 transcript levels, but not Lrs2 levels, are reduced in a *hfq* mutant, possibly due to reduced transcript stability (Figure 7C). These results point toward an overlapping regulatory network that includes a transcriptional regulatory cascade and a sRNA-regulated post-transcriptional regulatory component. The quorum sensing regulatory system therefore very likely integrates, in addition to cell density, a number of regulatory inputs and directs these to the most optimal responses for survival in a particular environment.

To further investigate the possible roles of Lrs1, we constructed an isogenic deletion mutant lacking the *lrs1* sequence. We first determined the precise 5′ and 3′ ends of the transcript following intramolecular ligation and synthesis of cDNA across the junction. The sequence of the cDNA was used to design PCR primers for deletion of the entire *lrs1* in *P. aeruginosa* PA14. Surprisingly, the culture of the mutant strain was devoid of the characteristic blue-green pigment of *P. aeruginosa*, a color that stems from production of pyocyanin (Figure S4C). Measurements of levels of pyoverdine, the fluorescent pigment produced by *P. aeruginosa*, showed no detectable differences between culture supernatants of wild-type and the *lrs1* mutant (data not shown). To detect the transcriptional changes that might contribute to this phenotype we constructed additional RNA-seq libraries from wild type and Δ*lrs1* isolates. Interestingly, the gene expression in both libraries was almost identical (Pearson r>0.99), except for a single operon, the anthranilate dioxygenase operon (*antABC*), which was upregulated by more than 2-fold in the mutant strain. This might explain a highly efficient metabolic flux to this pathway, which in turn depleted the production of pyocyanin, due to lack of chorismic acid (Figure S1); in turn this may provide an explanation for the lack of the color in the Δ*lrs1* cultures. Strikingly, Δ*lrs1* also resulted in a significant (over 4-fold) upregulation in the expression of the two PrrF sRNAs, which are regulators of a number of iron-related genes [69].

### The *Pseudomonas* transcriptome browser

As part of this study, we created a comprehensive graphical transcriptome map of *P. aeruginosa*. This map provides a high-resolution view of the *Pseudomonas* transcriptome structure and its regulatory sequences. To make this data an accessible resource for further research we generated an online repository and a data-viewer, The *Pseudomonas* transcriptome browser, which is available at http://www.weizmann.ac.il/molgen/Sorek/pseudomonas_browser/. The transcriptome browser allows navigating the transcriptome and genome data, and provides direct links to additional *Pseudomonas* resources.

In conclusion, the high-resolution analysis of the transcriptome of the opportunistic pathogen *P. aeruginosa* presented here provides a framework for studying the regulatory mechanisms that allow this common soil organism to become a successful human pathogen in various compromised hosts. In this work, we provide a detailed snapshot of the operon organization, the abundance of coding and non-coding transcripts and their sites of transcription initiation, taken at temperatures encountered most likely in the natural environment and in the human body. Taking advantage of this data, we could significantly expand the number of genes that are under the direct control of LasR, the master quorum-sensing regulator, including two novel sRNAs. In addition to creating a valuable tool for the *P. aeruginosa* research community, *The Pseudomonas transcriptome browser*, our work demonstrates an important function for temperature in controlling the expression of many of *P. aeruginosa* virulence factors. Considering the wide range of reservoirs, including the human body, this detailed transcriptome analysis should provide insights into the interactions between the various mechanisms that direct the assimilation of various signals and directing selective and coordinated gene expression to favor survival of this organism in often challenging environments.

## Materials and Methods

### Strains, mutant construction and culture conditions

*P. aeruginosa* PA14 and PAO1 are standard laboratory strains. *P. aeruginosa* PAK Δ*pscC* was described previously [70]. Unmarked mutations in *P. aeruginosa* PA14 in *lrs1* and *hfq* were generated using pEXG2 and the sucrose counter-selection method [71]. The pET29a-hfq recombinant plasmid was constructed by PCR amplification of the 249 bp coding sequence of *P. aeruginosa hfq* gene and cloning the DNA fragment into pET29a vector. *P. aeruginosa lasR* and *P. aeruginosa rhlR* mutant strains were constructed by introducing pJTT4 and pEXG2-RhlR into *P. aeruginosa* PA14 as previously described [72]. The complementation plasmid pLasR was constructed by PCR amplification of the *lasR* coding sequence and cloning the amplicon into pMMB67EH [73]. Strains defective in type III secretion system proteins and effectors were described previously [74]. *E. coli* and *P. aeruginosa* strains were grown in Luria broth (LB; Lennox formulation) unless otherwise noted. For monitoring the expression of the type III secretion effectors ExoS, ExoU, and the secretin PscC, the MinS medium lacking calcium supplement was used [75].

### RNA harvesting and cDNA synthesis

RNA was isolated from *P. aeruginosa* as described previously [76]. Briefly, bacteria were grown in LB to early stationary phase [optical density at 600 nm (O.D._600_) of 1.8] and total RNA was isolated from ∼7×10^8^ cells using hot phenol followed by ethanol precipitation. The samples were digested with DNase I for 2 hr, followed by phenol-chloroform-isoamyl alcohol extraction and ethanol precipitation. The precipitated sample was resuspended in 20 µL of RNase-free water. The quality of the resultant RNA was determined using an Agilent Bioanalyzer. For samples with RNA Integrity Numbers of greater than 7.5, the MICROBExpress Kit (Ambion) was used to deplete rRNA from the total RNA samples. The loss of intact rRNA was verified using the Agilent Bioanalyzer. cDNA was generated by using the methods from the Superscript Double-Stranded cDNA Synthesis Kit (Invitrogen) per manufacturer's instructions.

### Library preparation and sequencing

Illumina RNA-seq whole-transcriptome libraries were prepared using the Paired End Sequencing Sample Preparation Guide (Illumina) per the manufacturer's instructions as previously described [15], [77].

RNA from the same extractions was subjected to the strand-specific 5′-end sequencing protocol as previously described [17]. Briefly, RNA samples of both conditions were divided into two sub-samples (hereby called TAP+ and TAP−) containing 2.5 µg from rRNA-depleted RNA. The RNA of TAP+ sub-sample was incubated with 2 U of tobacco acid pyrophosphatase (TAP, Epicentre) for 1.5 hr at 37°C to generate 5′ monophosphate RNAs. The other sub-sample (TAP−) was not treated with TAP. cDNA sequencing libraries were prepared as previously described [15], [17], [77] with an addition of a unique 4-base index sequence to the 5′ RNA adapter used for each sub-sample followed by 2 random bases for ligation quality control. cDNA libraries of every two sub-samples were mixed in equal amounts prior to Illumina sequencing. The resulting sequences were sorted to the correct sub-sample by their index bioinformatically.

### Read mapping and identification of differentially expressed genes

Sequencing reads were mapped to the corresponding reference genome (Genbank: NC_008463) as described previously [15], [17], [77]. The number of overlapping mapped reads was counted for every ORF in each sample of whole-transcriptome sequencing (2 biological replicates for the 28°C and 37°C samples). For each ORF the number of reads per kilobase of gene model per million mapped reads (RPKM) was calculated as previously described by Mortazavi et al. [78]; this procedure also normalizes samples having different numbers of initial reads. Differentially expressed genes were called by performing χ^2^ test on the RPKM values and applying Bonferroni correction for multiple-testing. Genes with adjusted p-values smaller or equal to 0.05 in both replicates were determined to be differentially expressed.

### Gene ontology enrichment assay and determination of virulence related genes

Gene ontology (GO) annotation for *Pseudomonas aeruginosa* PAO1 was downloaded from the Gene Ontology repository website (http://cvsweb.geneontology.org/cgi-bin/cvsweb.cgi/go/gene-associations/gene_association.pseudocap.gz). Gene identifications (gene ids) for strain PAO1 were substituted with homolog gene ids for strain PA14 (homologs were downloaded from http://www.pseudomonas.com/download.jsp). Differentially expressed genes that have homologs in strain PAO1 (224/234 and 137/144 for 28°C and 37°C samples, respectively) were tested for GO enrichment against the entire set of ORFs of PAO1, which have homologs in PA14 (n = 5374) using Ontologizer [79]. GO categories with adjusted p-values smaller than 0.05 were considered significant. A list of virulence-related genes in *P. aeruginosa* was downloaded from the Virulence Factors Database (VFDB) (http://www.mgc.ac.cn/VFs/) and genes in the list were considered to be virulence-related for subsequent analyses.

### Determination of TSSs

For each position in the genome the number of mapped reads derived from the 5′ end sequencing experiments was recorded in a strand-specific manner. Positions with five or more uniquely mapped reads (encompassing 78% of the reads) were considered as reproducible sites. Reproducible sites in intergenic regions or overlapping ORFs were associated with the closest gene according to the gene annotation (Genbank: NC_008463). In cases where the site was positioned over 250 nt from the downstream gene the site was not associated with any gene. All reproducible sites that were not associated with genes were inspected manually using the *Pseudomonas transcriptome browser* and in cases which had a continuous whole-transcriptome expression from the site to the beginning of a downstream gene, the site was associated with the corresponding gene. The TSS of each gene was defined as the reproducible site upstream to the beginning of the gene with the highest number of reads in comparison to other sites that were associated with the same gene, as previously described [15], [17]. This was validated by randomly selecting 200 TSSs and checking for their agreement with the whole-transcriptome sequencing data, searching for continuous expression from the TSS in the direction of the transcription. TSSs (n = 2,117) were further validated by comparing the ratio of reads derived from the TAP+ sample divided by the number of reads from TAP− sample. The TAP+/TAP− ratio of putative TSSs were found to be enriched by an average of 2.6-fold (Wilcoxon sum rank p<1^−193^) as compared to non-TSS sites, indicating that they were preceded by a triphosphate, a hallmark of primary transcripts [80].

### Generation of transcriptional unit map

Genes that begin a transcriptional unit (TU) were defined as (1) genes with a defined TSS; (2) genes with an upstream gene located on the opposite strand; or (3) expressed genes that lacked a defined TSS were defined as beginning a TU, if the upstream gene was not expressed, or was expressed by at least 2-fold difference. When genes were not expressed under the conditions tested, operon structures were determined based on distance between pairs of genes, where genes occurring on the same strand with a distance shorter than 72 nt considered as belonging to the same operon. This threshold was empirically determined based on the distances between genes in expressed polycistrons with defined TSSs (n = 646; median distance = 10 nt), where the 90th-percentile distance between genes was 72 nt.

### Genome-wide detection of sRNAs

Small RNAs were identified based on the presence of a TSS and expression in intergenic regions (IGRs) that was not associated with genes. The TSS site was marked as the 5′ end of the putative sRNA and the 3′ end was estimated from the whole-transcriptome data (Table S6). Previously described sRNAs in *Pseudomonas aeruginosa* PA14 that were expressed in the 5′ end-sequencing and/or the whole-transcriptome data were included in the list. In addition, the sequence of known sRNAs from the *P. aeruginosa* PAO1 were searched for in the PA14 genome by a BLAST search (parameters ‘-p blastn –e 0.001 –F F –W 4 –q 1’) and sRNAs that were found and expressed in the PA14 genome were also included in the list. All expressed regions in the list were examined manually in the *Pseudomonas transcriptome browser* and were validated to be transcribed independently from their downstream genes; putative sRNAs that were not transcribed independently (i.e., transcribed along with the downstream or upstream gene) were removed from the list. All remaining intergenic transcripts represented independent expression of either a non-coding RNA or an un-annotated ORF. Therefore, all intergenic transcripts were scanned for putative ORFs and the longest possible ORF lacking a stop-codon in every region of expression was subsequently searched for homology against the entire protein collection (nr) using BLAST+ with a minimal e-value of 10^−4^ [81]. Putative ORFs with homologs in other organisms were removed from the list and moved to a list of un-annotated ORFs in *P. aeruginosa* PA14 genome (Table S5). sRNAs containing putative ORFs larger than 50 amino acids and lacking sequence homology in the protein database were marked as putative ORFs in the list of sRNAs. Terminator sequences for the sRNAs were predicted using TransTermHP v2.09 [82].

The conservation of the sRNAs was tested using BLASTN search against the entire nucleotide collection (nt) with the following parameters (‘-W 4 –e 0.001 –F F –q 1’) demanding that homologs would span at least 50% of the sRNA sequence. BLAST reports were analyzed and each sRNA was assigned a category as follows: (1) PA14-specific when homologs were not identified; (2) *P. aeruginosa-*specific when homologs were found only in the *P. aeruginosa* lineage; (3) *Pseudomonas-*specific when homologs were found also in other *Pseudomonas* species; and (4) widespread when the sRNA sequence was found outside of the *Pseudomonas* lineage.

### Computational identification of LasR sites in gene promoters

The sequences of promoters known to be directly regulated by LasR were downloaded from the Prodoric database [83]. The sequences upstream to all defined TSSs of ORFs and ncRNAs (200 nt) were extracted from the PA14 genome and searched for significant matches to the PSSM model using matrix-scan [84] with the following parameters (‘–pseudo 1 -decimals 1 -1str -origin end -bginput -markov 0 -bg_pseudo 0.01 -return sites -lth score 0’). The process was performed iteratively to refine the PSSM for 3 rounds until convergence of the matches was achieved (Table S4).

### Quantitative Real-Time PCR

Unique primers were designed for ∼100 bp and ∼400 bp segments of transcripts using MacVector software. Triplicate qRT-PCR reactions were set up using 1X concentration of SYBR FAST One-Step qRT-PCR Universal (Kapa Biosystems), optimized concentrations of appropriate primers, and one of the following templates: 10^8^, 10^6^, 10^5^, 10^4^, 10^3^, 10^2^ molecules of ∼400 bp PCR-generated standard curve template, 20 ng of the total RNA used for Illumina sequencing, 80 ng of genomic DNA, or water (no template control). Reactions were run in a Mastercycler realplex RT-PCR machine (Eppendorf) and the PCR was performed with the following conditions: 42°C for 5 min, 95°C for 5 min, (95°C for 3 sec, 60°C for 20 sec)×40 cycles. After determining the absolute number of copies in each sample for each gene, relative concentrations were calculated using Microsoft Excel.

### Western immunoblotting

Wild-type and the *pscC*, *exoU* and *exoS* mutants of *P. aeruginosa* were grown from fresh overnight cultures to an O.D._600_ of 1.8. Cells were collected from 1 mL aliquots by centrifugation. Five hundred microliters of supernatants were concentrated 20-fold using an Amicon Ultra 10 kDa MW limit column (Millipore). Cell pellets and concentrated supernatants were dissolved in 4X SDS-PAGE buffer and normalized according to their optical density at time of harvesting. Equal volumes of each were loaded on 8–10% SDS polyacrylamide gels, electrophoresed, and transferred to PVDF membranes for Western immunoblotting. Blots were blocked in 5% non-fat skim milk, and incubated with the following dilutions of the primary antibodies, as described (Hoang et al., 2001): anti-PscC at 1∶100, anti-ExoS and anti-OprF at 1∶5000 and anti-ExoU (generously provided by Dr. Dara Frank, Medical College of Wisconsin) at 1∶10,000. Blots were washed with 1X PBST, followed with probing by goat anti-rabbit-HRP conjugate antibodies (BioRad). After washing again, blots were visualized following treatment by SuperSignal West Pico Chemiluminescent Substrate (Thermo Scientific).

### Determination of 5′ and 3′ ends of transcripts following intramolecular ligation for *lrs1*

The procedure for mapping the 5′ and 3′ ends of transcripts was carried out as previously described [85] with minor modifications. Total RNA was isolated from early stationary phase of *P. aeruginosa* PA14 and residual DNA was removed with TURBO DNase (Ambion). Total RNA (6.5 µg) was treated with 5 U of tobacco acid pyrophosphatase (Epicentre) for 1 hr at 37°C. Following phenol/chloroform extraction and ethanol precipitation, RNA was treated for 16 hr at 16°C with 20 U of T4 RNA ligase (NEB) for RNA circularization. After phenol/chloroform extraction and ethanol precipitation, 1.5 µg of self-ligated RNA was reverse transcribed using an *lrs1* specific primer (R_lrs1+151; Table S7) and SuperScriptIII First-Strand Synthesis system (Invitrogen). RNA was removed with 25 U of RNase If (NEB) in addition to RNase H treatment after termination of the reaction. Approximately 10% of the reaction was used as the template for PCR amplification using GoTaq Green Master Mix (Promega) and the lrs1 primer pairs of R_lrs1+126/F_lrs1+152. Cycling conditions were: 94°C/3 min; 35 cycles of 94°C/45 sec, 61°C/30 sec, 72°C/30 sec; 72°C/3 min. The PCR products were separated by agarose gel electrophoresis; DNA bands were eluted and cloned into pCR2.1 TOPO vector (Invitrogen) and the inserts were sequenced using the M13R primer.

### RNA isolation and Northern blot analysis

Overnight cultures grown at 37°C were diluted in LB containing gentamicin (75 µg/mL) to an initial O.D._600_ ∼0.02. Cell growth was monitored at each time point and IPTG was added at the defined time points (4, 8, and 12 hr) at 2 mM final concentration. After IPTG induction for 30 min, total RNA was isolated by the hot phenol method as previously described [76]. Total RNA (5 µg) was fractionated on a 5% polyacrylamide gel/7M urea gel and transferred onto a Hybond-XL membrane (GE Healthcare). RNAs were UV cross-linked to the membrane and hybridized at 42°C with oligonucleotides R_lrs1+126 and R_5S+67 as probes for Lrs1 and 5S rRNA, respectively, and then 5′-end radiolabeled using [γ-^32^P]-ATP (Perkin Elmer) and T4 polynucleotide kinase (NEB). Signals were visualized with a phosphoimager (Typhoon 9400, GE Healthcare) and analyzed with ImageQuant TL software (GE Healthcare).

### Electrophoretic mobility shift assays (EMSA)

We assessed LasR binding to PCR-generated DNA using EMSA as previously described [86]. PCR products were end-labeled with [γ-^32^P]-ATP (Perkin Elmer) with T4 polynucleotide kinase (NEB). Binding reactions contained 1 pM of both a specific and nonspecific probe and 30, 3, or 0.3 pM purified LasR in a total volume of 20 µLDNA binding buffer (20 mM Tris-HCl [pH 7.5], 50 mM KCl, 1 mM EDTA, 1 mM DTT, 100 µg/ml bovine serum albumin, 10% glycerol). For each DNA target, a control reaction contained probe but no LasR. Mixtures were incubated for 20 min at room temperature and separated on a non-denaturing 5% Tris-glycine-EDTA polyacrylamide gel for 120 min at 50 V using a mini-PROTEAN tetra-cell (Bio-Rad). The gels were dried and exposed to a storage phosphor screen; exposure was visualized using a Storm phosphorimager and ImageQuant TL software (GE Healthcare).

### Hfq gel shift assay

Hfq was purified from lysates of *E. coli* BL21(DE3) pLysS cells harboring pET29a-*hfq* grown in LB medium at 37°C. Expression of the protein was induced by the addition of IPTG to 1 mM when the cultures were at O.D._600_ of 0.4. After an additional 3 hr incubation at 37°C, cells were collected and lysed by sonication in 30 mL of buffer A (50 mM sodium phosphate, 300 mM NaCl) and a protease inhibitor cocktail tablet (Complete mini, EDTA-free, Roche Diagnostic) was added. Cells were treated with 20 U of TURBO DNase (Ambion) and 100 µg of RNase A and incubated on ice for 1 hr. Centrifugally-clarified lysate was passed over a Co^2+^ affinity column (Clontech). Eluted fractions was pooled and dialyzed with buffer B (10 mM Tris-Cl [pH 7.5], 50 mM NH_4_Cl, 0.2 mM EDTA, 2% glycerol).

DNA templates for *in vitro* transcription of *lrs1* and *prrF1* were generated by PCR using genomic DNA with primer pairs of F_T7lrs1+1/R_lrs1+191 and F_T7prrf1+1/R_prrf1+116 (Table S7), respectively. Transcription reactions were carried out using MEGAscript (Ambion). Gel-purified *lrs1* transcripts were 5′ end labeled with [γ-^32^P]-ATP using T4 polynucleotide kinase (NEB). Labeled *lrs1* transcripts (0.1 nM) were incubated with the purified Hfq protein in 20 µL of HB buffer (10 mM Tris-Cl pH 7.5, 50 mM NH_4_Cl, 0.2 mM EDTA, 10% glycerol) for 15 min at 25°C as described previously [87]. The reaction mixtures were then analyzed on 5% polyacrylamide gels.

### Construction of *lacZ* fusions and β-galactosidase assays

Promoter fragments including the putative LasR binding sites were amplified by PCR from *P. aeruginosa* PA14 genomic DNA using each specific primer pairs shown in Table S7. Each fragment was cloned into the integration vector mini-CTX-lacZ. These constructs and the vector control were integrated into the chromosome of *P. aeruginosa* PA14 as described previously [88]. The assay for activation of transcription by homoserine lactones in *E. coli* was carried out as described previously [72]. The promoter-containing fragments of *phzA1*, *phzM*, and *rhlA* were cloned into pZE21-LacZ and transformed into strains harboring plasmids expressing *lasR*, *rhlR*, or an empty vector. β-galactosidase activities were assayed as previously described [72].

### Pyocyanin assay

Pyocyanin was extracted from 1 mL of culture supernatant following addition of 0.6 mL chloroform. After extraction, the organic phase was removed and added to 1 mL of 0.2 N HCl. Following centrifugation, 0.8 mL of the aqueous phase was removed. Concentration of pyocyanin was determined by multiplying the absorbance at 520 nm by 30 (the dilution factor) and then by 17.072 [46].

### PQS assay

To quantify PQS levels, a bioassay was developed where a *lacZ* fusion responds to exogenous PQS. A *pqsA-lacZ* transcriptional fusion was constructed by cloning a DNA fragment corresponding to *pqsA* (−246 to +231 relative to the start site of transcription) into mini-CTX-lacZ and conjugating it into PAO1Δ*pqsA* strain. The β-galactosidase activities in this strain were measured by addition of supernatants from culture grown at two different temperatures and at two different growth phases. For supernatant isolation, the equal amounts of diluted PA14 (O.D._600_ ∼1.0) grown overnight at 28°C and 37°C were diluted 1∶100 in 25 mL LB medium and cultured to early stationary and stationary phase by monitoring the O.D._600_. Next, 1 mL of the culture was isolated by centrifugation at 10,000×g for 5 min and the supernatant was passed through a 0.2 µm syringe filter. For the bioassay, the reporter strain (PAO1 *pqsA-lacZ* Δ*pqsA*) was grown overnight at 37°C and diluted to 1∶200 in 10 mL LB medium containing 10 µg/ml tetracycline. The culture was incubated at 37°C until it reached an O.D._600_ of 1. One hundred µL of the reporter strain were mixed with 100 µL of each supernatant and then incubated at 37°C for 30 min. β-galactosidase activities were assayed as previously described [72].

### Pyoverdine assay

Pyoverdine was measured by growing the bacteria in King's B medium. Strains grown on King's B agar were cultured in 2 mL liquid King's B medium at 37°C overnight. Cultures were diluted to an initial O.D._600_ at ∼0.05 in 25 mL King's B liquid medium and then incubated at 37°C with shaking. At defined time points, the O.D._600_was measured and supernatants were isolated from 1 mL of each culture by centrifugation at 1,000×g for 3 min. The absorbance (A) at 400 nm was measured and expressed as the ratio of A_400_/A_600_.

## Supporting Information

Figure S1 **Effects of temperature on the enzymes of various metabolic pathways providing precursors of quinolones, pyochelin, phenazine and TCA cycle intermediates.** Enzymes whose transcript levels were increased in *P. aeruginosa* grown at 37°C are boxed in red; those that were decreased at this temperature are boxed in blue. Transcripts for enzymes in black boxes and crossed boxes were unchanged or not detected, respectively.
(TIF)

Figure S2 **The transcriptome structure of** ***P. aeruginosa*** **PA14.** (A) Number of genes in TUs. Shown is a histogram of the numbers of genes per TU. The vast majority of genes are found in mono- and bi- cistrons, and only a minority of the genes is transcribed as longer operons. (B) Length distribution of 5′ UTRs in *P. aeruginosa*. Only a small number of the *P. aeruginosa* 5′ UTRs are longer than 100 nt (median length of 47 nt). The unbiased TSS mapping allowed the detection of a small group of genes with extraordinarily long 5′ UTRs (>250 nt), which might represent *cis*-regulatory sequences.
(TIF)

Figure S3 **LasR-dependent expression of LacZ reporter fusions.** The same fragments used in the EMSA (Figure 3C) were cloned upstream of a *lacZ* gene and introduced into *P. aeruginosa* PA14 wild-type and Δ*lasR*. Overnight cultures grown at 37°C were diluted in LB to an initial O.D._600_ of 0.02. The β–galactosidase activity was measured in cultures at (A) late logarithmic (8 h) and (B) stationary phase (12 h) of growth. A two-tailed student's t-test assuming equal variance on the three replicates of wild-type and mutant strains was used to evaluate the significance of the differences in β–galactosidase activity between samples. Asterisks indicate p-values less than 0.01 between wild-type and mutant strains.
(TIF)

Figure S4 **Expression and regulation of pigment production by Lrs1.** Shown are growth (A) and Northern blot analysis of Lrs1 expression (B) in (i) *P. aeruginosa* PA14 carrying an empty vector; (ii) a PA14 Δ*lasR* strain carrying an empty vector, and (iii) a PA14 Δ*lasR* strain containing the complementation plasmid pLasR. Northern blot performed separately on the 3 strains validated the expression of the Lrs1 sRNA in strains expressing LasR either from chromosomal or extra-chromosomal origins. (C) Absence of the green pigment from a culture of an *lrs1* mutant (left), compared to wild-type *P. aeruginosa* PA14 grown for 8 hours at 37°C.
(TIF)

Table S1 **Summary of short-read sequencing.**
(DOCX)

Table S2 **Genes with altered mRNA levels when** ***P. aeruginosa*** **PA14 is grown at 37°C compared to 28°C.**
(XLSX)

Table S3 **Operon organization and definition of transcriptional units.**
(XLSX)

Table S4 **LasR binding sites.**
(XLSX)

Table S5 **Unannotated open reading frames in** ***P. aeruginosa*** **PA14 found in this study.**
(XLSX)

Table S6 **Intergenic non-coding RNAs.**
(XLSX)

Table S7 **Sequences of primers used for PCR amplification of LasR regulated promoter-containing fragments.**
(DOCX)
