# Supplementary figures and images for: The Single-Nucleotide Resolution Transcriptome of Pseudomonas aeruginosa Grown in Body Temperature
Source: PLoS Pathog. 2012 Sep 27;8(9):e1002945. doi: 10.1371/journal.ppat.1002945 (PMC3460626; doi:10.1371/journal.ppat.1002945)

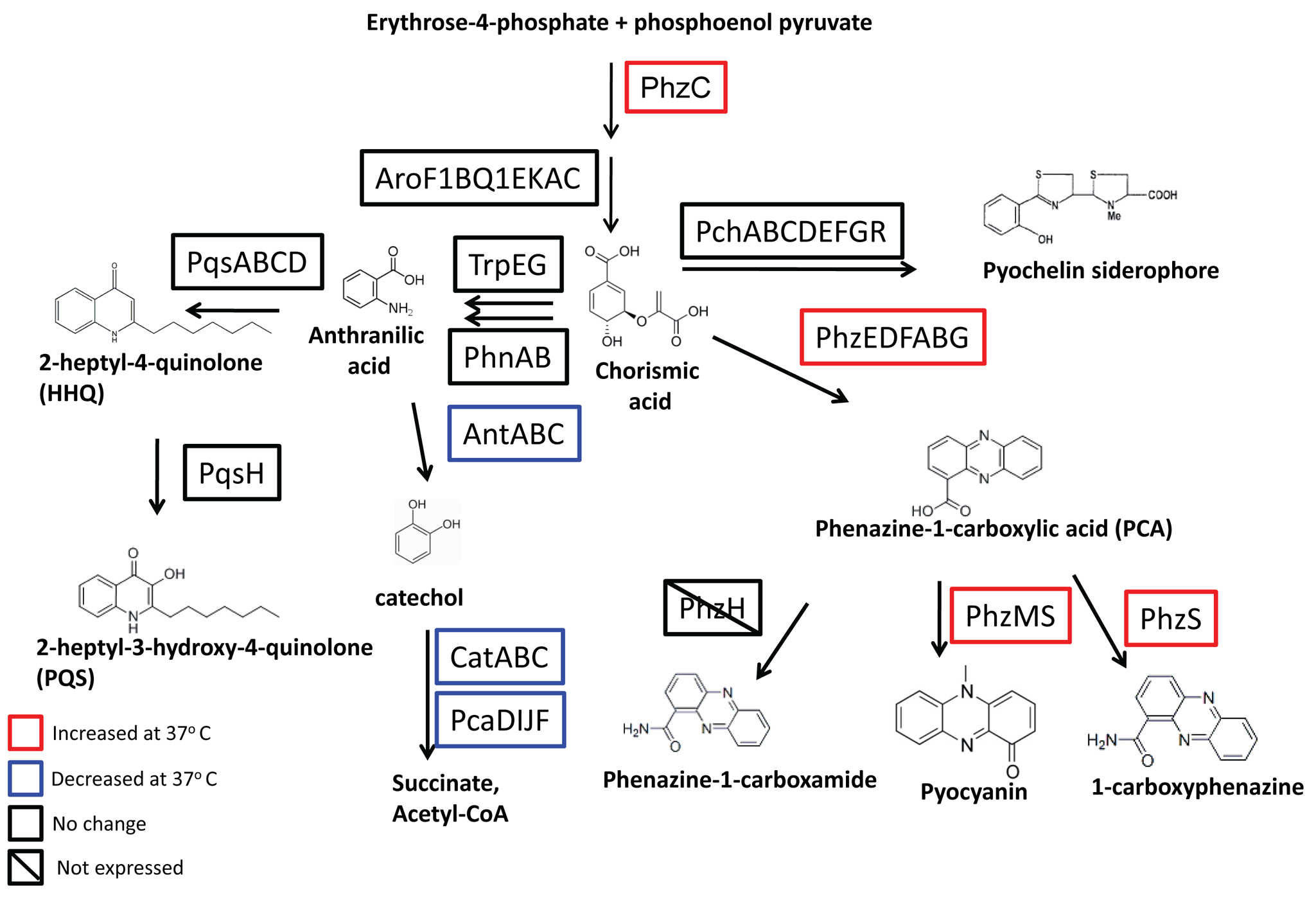

Supplement: Figure S1 — Effects of temperature on the enzymes of various metabolic pathways providing precursors of quinolones, pyochelin, phenazine and TCA cycle intermediates. Enzymes whose transcript levels were increased in P. aeruginosa grown at 37°C are boxed in red; those that were decreased at this temperature are boxed in blue. Transcripts for enzymes in black boxes and crossed boxes were unchanged or not detected, respectively. (TIF) [file ppat.1002945.s001.tif]

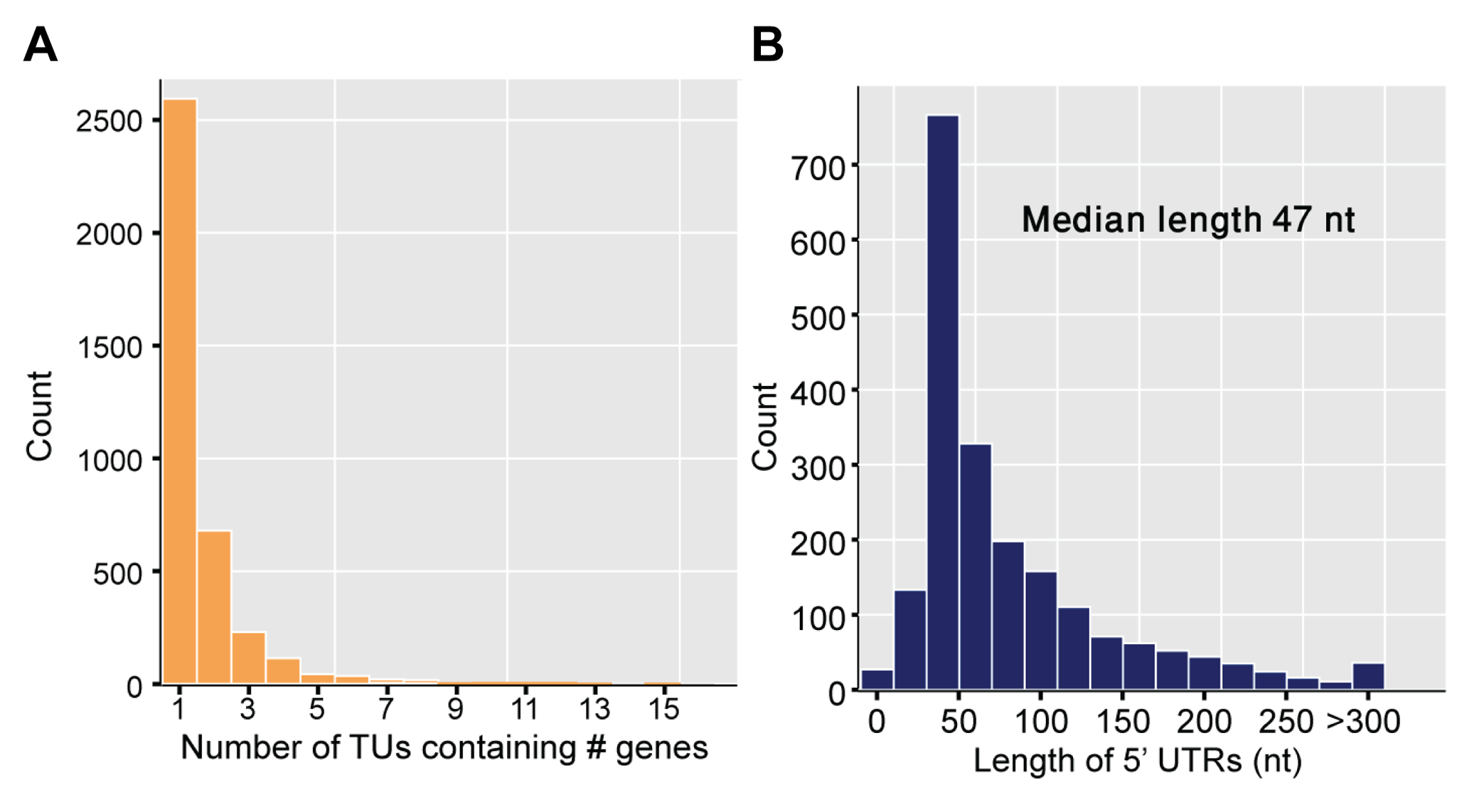

Supplement: Figure S2 — The transcriptome structure of P. aeruginosa PA14. (A) Number of genes in TUs. Shown is a histogram of the numbers of genes per TU. The vast majority of genes are found in mono- and bi- cistrons, and only a minority of the genes is transcribed as longer operons. (B) Length distribution of 5′ UTRs in P. aeruginosa. Only a small number of the P. aeruginosa 5′ UTRs are longer than 100 nt (median length of 47 nt). The unbiased TSS mapping allowed the detection of a small group of genes with extraordinarily long 5′ UTRs (>250 nt), which might represent cis-regulatory sequences. (TIF) [file ppat.1002945.s002.tif]

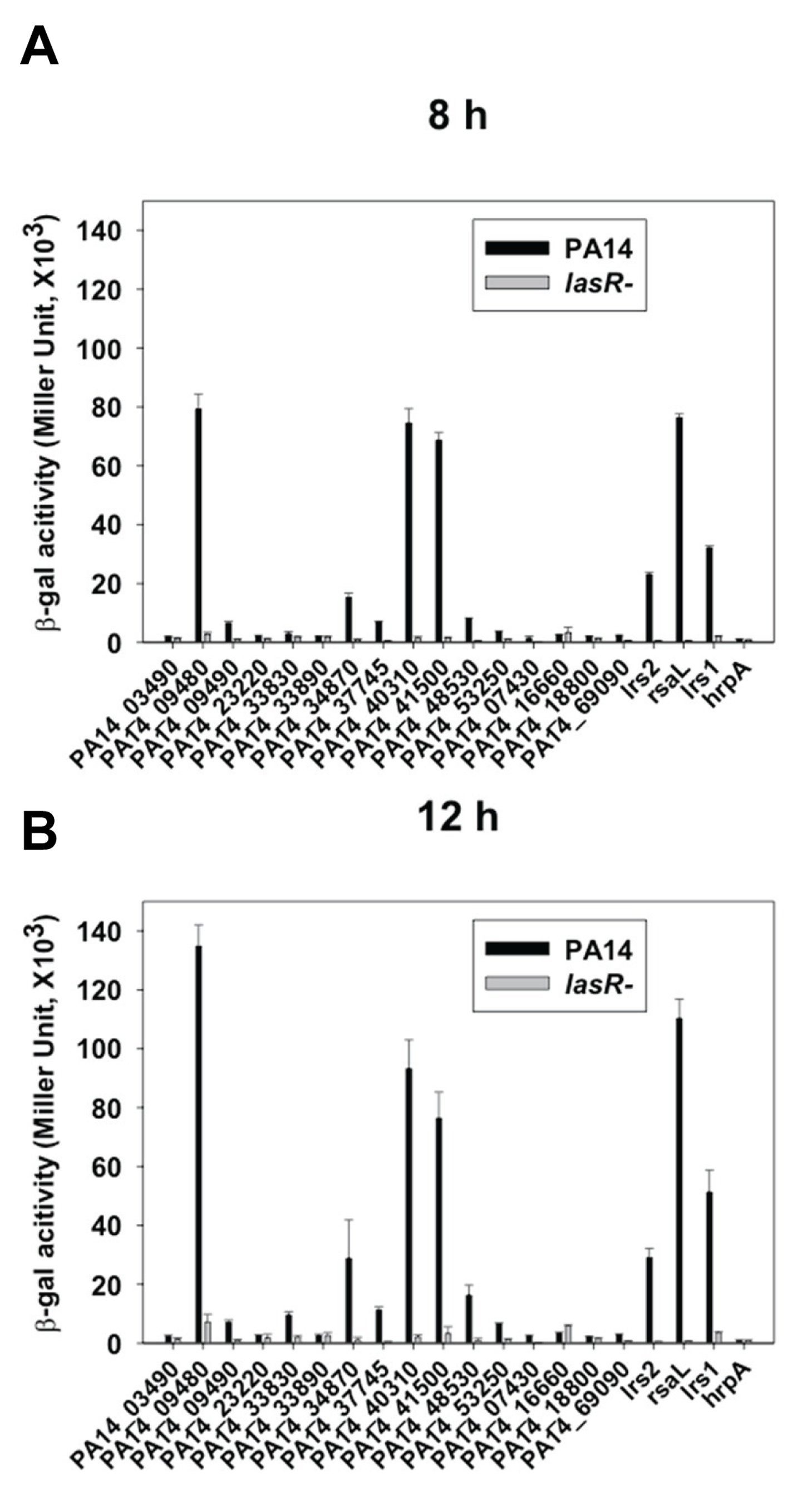

Supplement: Figure S3 — LasR-dependent expression of LacZ reporter fusions. The same fragments used in the EMSA (Figure 3C) were cloned upstream of a lacZ gene and introduced into P. aeruginosa PA14 wild-type and ΔlasR. Overnight cultures grown at 37°C were diluted in LB to an initial O.D.600 of 0.02. The β–galactosidase activity was measured in cultures at (A) late logarithmic (8 h) and (B) stationary phase (12 h) of growth. A two-tailed student's t-test assuming equal variance on the three replicates of wild-type and mutant strains was used to evaluate the significance of the differences in β–galactosidase activity between samples. Asterisks indicate p-values less than 0.01 between wild-type and mutant strains. (TIF) [file ppat.1002945.s003.tif]

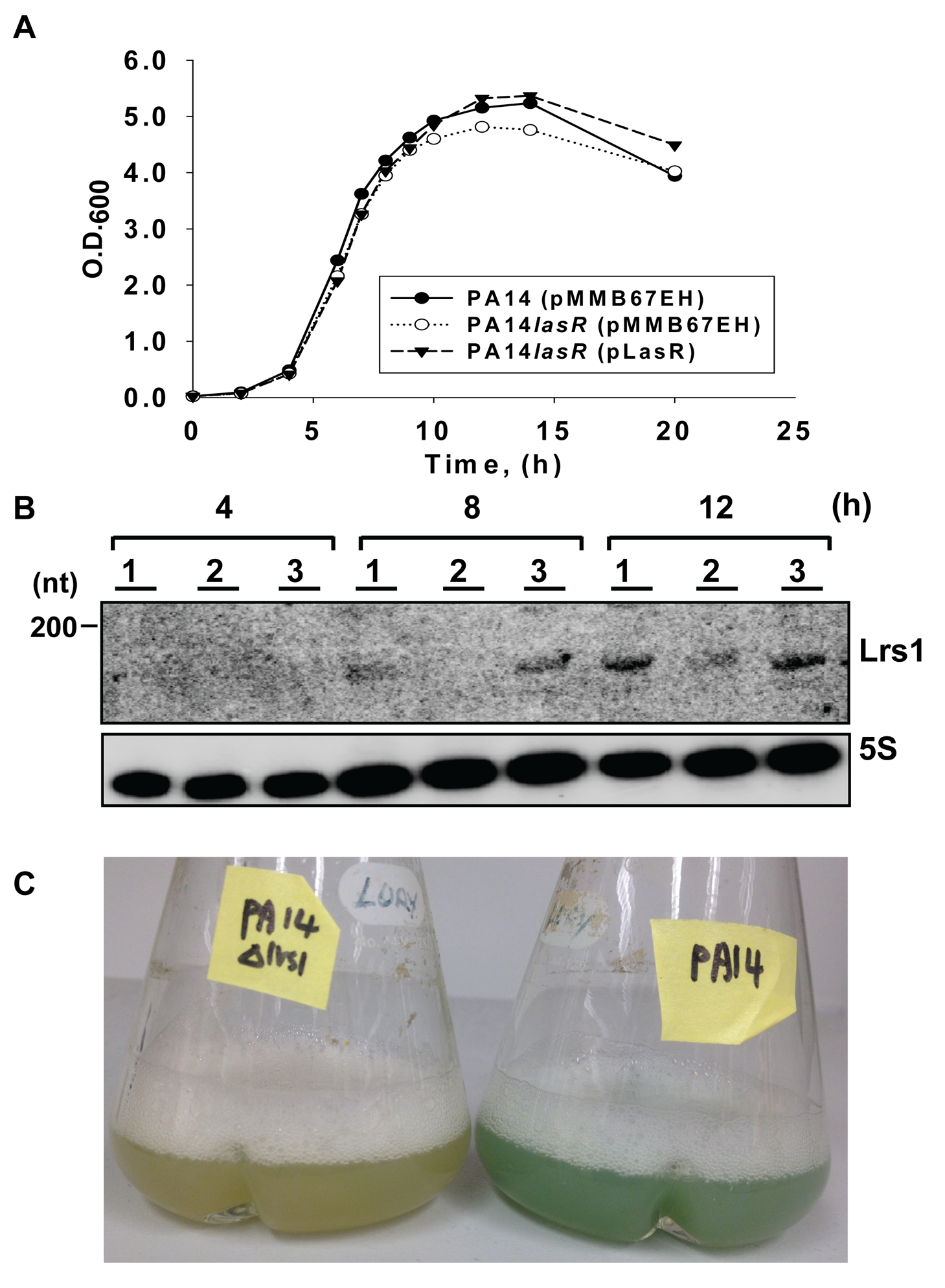

Supplement: Figure S4 — Expression and regulation of pigment production by Lrs1. Shown are growth (A) and Northern blot analysis of Lrs1 expression (B) in (i) P. aeruginosa PA14 carrying an empty vector; (ii) a PA14 ΔlasR strain carrying an empty vector, and (iii) a PA14 ΔlasR strain containing the complementation plasmid pLasR. Northern blot performed separately on the 3 strains validated the expression of the Lrs1 sRNA in strains expressing LasR either from chromosomal or extra-chromosomal origins. (C) Absence of the green pigment from a culture of an lrs1 mutant (left), compared to wild-type P. aeruginosa PA14 grown for 8 hours at 37°C. (TIF) [file ppat.1002945.s004.tif]
