# Supplementary material for: The Single-Nucleotide Resolution Transcriptome of Pseudomonas aeruginosa Grown in Body Temperature
Source: PLoS Pathog. 2012 Sep 27;8(9):e1002945. doi: 10.1371/journal.ppat.1002945 (PMC3460626; doi:10.1371/journal.ppat.1002945)
Supplement: Table S1 — Summary of short-read sequencing. (DOCX) [file ppat.1002945.s005.docx]

| **Protocol** | **Whole-transcriptome sequencing** | | | | **5' end sequencing** | | | |
| --- | --- | --- | --- | --- | --- | --- | --- | --- |
| Condition | **28^o^C** | | **37^o^C** | | **28^o^C** | | **37^o^C** | |
| Sample | Replicate 1 | Replicate 2 | Replicate 1 | Replicate 2 | TAP+* | TAP-* | TAP+ | TAP- |
| Number of reads | 14,906,429 | 44,653,892 | 15,039,623 | 33,393,860 | 18,937,154 | 13,369,178 | 4,097,695 | 1,866,801 |
| Mapped reads | 14,868,477 | 35,456,044 | 15,000,121 | 29,123,620 | 10,047,862 | 9,730,899 | 2,240,093 | 1,375,268 |
| % mapped to non-rRNA regions | 14.2 | 16.7 | 19.1 | 13.2 | 12.56 | 7.89 | 9.75 | 8.94 |

*Indicates whether the RNA sample was treated (TAP+) or not treated (TAP-) with tobacco acid pyrophosphatase.
