# Supplementary material for: The Single-Nucleotide Resolution Transcriptome of Pseudomonas aeruginosa Grown in Body Temperature
Source: PLoS Pathog. 2012 Sep 27;8(9):e1002945. doi: 10.1371/journal.ppat.1002945 (PMC3460626; doi:10.1371/journal.ppat.1002945)
Supplement: Table S7 — Sequences of primers used for PCR amplification of LasR regulated promoter-containing fragments. (DOCX) [file ppat.1002945.s011.docx]

**TABLE S7. Primers used in this study**

| **Name** | **Sequence (5' to 3')** | **Use** |
| --- | --- | --- |
| F_E_ PA14_03490 | GTCCGAATTCGTGGAGGATCGCGTCGC | gel shift assay |
| R_B_PA14_03490 | GTCCGGATCCGGCTGATTGGCAGCC | gel shift assay |
| F_E_ PA14_09480 | GTCCGAATTCTTTCCTGCGTACCGAAAG | gel shift assay |
| R_B_PA14_09480 | GTCCGGATCCTTCCGTGAAGTGTTTCAAATAG | gel shift assay |
| F_E_ PA14_09490 | GTCCGAATTCCGTCATTCCGTGAAGTG | gel shift assay |
| R_B_PA14_09490 | GTCCGGATCCGAAAGAATAAAATTACAACT TGGC | gel shift assay |
| F_E_ PA14_23220 | GTCCGAATTCGCGAAGATCGCCGCCAG | gel shift assay |
| R_B_PA14_23220 | GTCCGGATCCGTTGAAGAGTGAAGCCTTTG | gel shift assay |
| F_E_ PA14_33830 | GTCCGAATTCGCAATGGCAGACCTTGC | gel shift assay |
| R_B_PA14_33830 | GTCCGGATCCTGCACGTGTTCCCTGG | gel shift assay |
| F_H_ PA14_33890 | GTCCAAGCTTAGCGGCGAGGAGCGG | gel shift assay |
| R_B_PA14_33890 | GTCCGGATCCAGTCGCACGGCGACTG | gel shift assay |
| F_E_ PA14_34870 | GTCCGAATTCCTGCTGTCCCCGGCAC | gel shift assay |
| R_B_ PA14_34870 | GTCCGGATCCGTCTACCGCAGGGCATTC | gel shift assay |
| F_E_ PA14_37745 | GTCCGAATTCCTTCTGATGCCGGCGCG | gel shift assay |
| R_B_ PA14_37745 | GTCCGGATCCTGCGCGAACGGGTGG | gel shift assay |
| F_E_ PA14_40310 | GTCCGAATTCCCGGGAAGCGCCCTGTG | gel shift assay |
| R_B_ PA14_40310 | GTCCGGATCCACTGCCCGGCCCTGG | gel shift assay |
| F_E_ PA14_41500 | GTCCGAATTCGCGGCTGAATCCGGCTG | gel shift assay |
| R_B_ PA14_41500 | GTCCGGATCCGCCTGCGGCTCCTAG | gel shift assay |
| F_E_ PA14_48530 | GTCCGAATTCCCGTGCTACCCCCGG | gel shift assay |
| R_B_ PA14_48530 | GTCCGGATCCGGTTTCCGAAGTGTTTCGC | gel shift assay |
| F_E_ PA14_53250 | GTCCGAATTCGGCGGCCCTTTGCCTG | gel shift assay |
| R_B_ PA14_53250 | GTCCGGATCCTTTCCAGAACCGGATTC | gel shift assay |
| F_E_ PA14_07430 | GTCCGAATTCTGGATCGTTTCGTCACAGG | gel shift assay |
| R_B_ PA14_07430 | GTCCGGATCCAATGCCGGACATACGCATG | gel shift assay |
| F_E_ PA14_16660 | GTCCGAATTCTTCGCGCTCGTAGTAGC | gel shift assay |
| R_B_ PA14_16660 | GTCCGGATCCGTTTGACGTGTTGGGTG | gel shift assay |
| F_E_ PA14_18800 | GTCCGAATTCTTGTTCTATATGTTCGAACTTGGC | gel shift assay |
| R_B_ PA14_18800 | GTCCGGATCCTGGTTTGTTGACTCCTG | gel shift assay |
| F_E_ PA14_69090 | GTCCGAATTCTCCAGCAGACCCTCGAG | gel shift assay |
| R_B_ PA14_69090 | GTCCGGATCCGCAGGTGTTGATGCAC | gel shift assay |
| F_E_ lrs2 | GTCCGAATTCCAATGAAAGATTTTCAGCCAAACG | gel shift assay, lrs2-lacZ |
| R_B_ lrs2 | GTCCGGATCCCGAGGCTCTCCAGAG | gel shift assay, lrs2-lacZ |
| F_E_ rsaL | GTCCGAATTCTTGTGCATCTCGCCCAGC | gel shift assay |
| R_B_ rsaL | GTCCGGATCCTCGGACGTTTCTTCGAG | gel shift assay |
| F_E_lrs1 | GTCCGAATTCTGGCAGGCGAGACGGG | gel shift assay, lrs1-lacZ |
| R_B_lrs1 | GTCCGGATCCGTCCGAACCCATGAGATG | gel shift assay, lrs1-lacZ |
| F_E_hrpA | GTCCGAATTCCGGGAAGCGCCCTTCCTTG | gel shift assay |
| R_B_hrpA | GTCCGGATCCCGGCCCTGGCGATACCC | gel shift assay |
| R_lrs1+126 | CTGCGCGGGATCCGAGAACGGCAAC | 5' and 3', Northern blot mapping |
| R_lrs1+151 | CGAGAAATTTGGCACACCCACC | 5' and 3', mapping |
| F_lrs1+152 | CGGTTTGGATCGCGCCGATTGTCGC | 5' and 3' mapping |
| F_T7lrs1+1 | GCGTAATACGACTCACTATAGGCCATCTCAT GGGTTCGG | *in vitro* transcription |
| R_lrs1+191 | CGGGCTTCGTAGGCCGCGACAATC | *in vitro* transcription |
| F_T7prrf1+1 | GCGTAATACGACTCACTATAGGAACTGGTCGCAGATCAG | *in vitro* transcription |
| R_prrf1+116 | AAAAAAAGACCCGGCAAAGTGCCGGGTCAAAAACCGTGATTAGCC | *in vitro* transcription |
| F_NdI_hfqO1+1 | GTCC CATATGTCAAAAGGGCATTCGCTAC | pET29a+Hfq |
| R_XhI_hfqO1+stp | GTCC CTCGAG AGCGTTGCCCGGCTCGG | pET29a+Hfq |
| F_XhI_phzA1 | GTCCGGTACCTTCCGTGAAGTGTTTCAAATAG | pZE21-phzA1 |
|  |  |  |
| R_kpnI_phzA1 | GTCCGGTACCCCTCCT TTCCGTGAAGTGTTTCAAATAG | pZE21-phzA1 |
| F_XhI_phzM-63 | GTCCCTCGAGCCGGCTCAACTACAAGATC | pZE21-phzM |
| R_kpnI_phzM | GTCCGGTACCCCTCCTGAAAGAATAAAATTACAACTTGGC | pZE21-phzM |
| F_XhI_rhlA | GTCCCTCGAG CTTATGCGCAGGCGACC | pZE21-rhlA |
| R_kpnI_rhlA | GTCCGGTACCCCTCCTCGAACAGGCAAACAGCTATC | pZE21-rhlA |
| F_BHI_rhlR-483 | GTCCGGATCCCACTGGGAGCCTTGCTG | pEXG2-RhlR |
| R_Hnd3_rhlR+497 | GTCCAAGCTTGCGTAGCGCGAAAGCTC | pEXG2-RhlR |
| F_rhlR_fusN | CGGCGCGTCGTGCAGTAAGCCCTGATCG | pEXG2-RhlR |
| R_rhlR_fusN | CGGCGCGTCGTGCAGTAAGCCCTGATCG | pEXG2-RhlR |
| F_q_lrs1 | GCCGCCCTTCTTGCTTG | Real time PCR |
| R_q_lrs1 | TTTGGCACACCCACCG | Real time PCR |
| F_q_lrs2 | CTGGAGAGCCTCGAAACATAGG | Real time PCR |
| R_q_lrs2 | GGCTGTTATTGGGGATGTTT CTTGG | Real time PCR |
| F_q_prrf1 | CGCGAGATCAGCCGGTAAGC | Real time PCR |
| R_q_prrf1 | AAACCGTGATTAGCCTGATGAGGAG | Real time PCR |
| F_q_rsmZ | GAACACGCAACCCCGAAGGATC | Real time PCR |
| R_q_rsmZ | CACTCTTCAGTCCCTCGTCATCATC | Real time PCR |
| R_5S +67 | CGTTTCACTTCTGAGTTCGGGAAGG | Northern blot |
| F_Hnd3_pqsA  -246 | GTCCGTCCAAGCTTGGCCTCGAGCAAGGGTTG | pCTX-*pqsA*-*lacZ* cloning |
| R_XmaI_pqsA  +231 | GTCCCCCGGGCGCCGGGCTTGAGCAG | pCTX-*pqsA*-*lacZ* cloning |

Table S8. Strains and plasmids used in this study

| Strain or plasmid | Relevant genotype or description | Source or reference |
| --- | --- | --- |
| *P. aeruginosa* |  |  |
| PA14 | Wild type |  |
| PA14*lasR* | Isogenic deletion strain constructed with pJTT4 | This study |
| PA14*lrs1* | Isogenic deletion strain constructed with pEXG2-lrs1 | This study |
| PA14*rhlR* | Isogenic deletion strain constructed with pEXG-rhlR | This study |
| PA14 *phzM* | PA14 phzM::MAR2xT7 (mutant 40343) | (1) |
| PA14 *pqsA* | Deletion of *pqsA* gene, | Gift of Dr. Laurence Rahme |
| *pqsA-lacZ* PAO1Δ*pqsA* | Chromosomal *pqsA-lacZ* transcriptional fusion in PAO1 Δ*pqsA* | This study |
|  |  |  |
| *E.coli* |  |  |
| DH5α | φ80d*lacZ*∆M15 ∆(*lacZYA-argF*)*U169 recA1 endA1 hsdR17*(r_K_^-^ m _K_^-^) *supE44 thi-1 gyrA relA1* | (2) |
| BL21(DE3)/pLysS | F^-^ *ompT hsdS_B_(r^-^_B_m^-^_B_ ) gal dcm* (DE3) pLysS(Cm^r^) | Novagen |
| SM10 | *thi-1 leuB6 supE44 tonA21 lacY1 recA*::RP4-2-Tc::Mu Km^r^ | (3) |
|  |  |  |
| Plasmids |  |  |
| pEXG2 | ColE1 suicide vector; mob sacB Gmr | (4) |
| pMMB67EH | Broad-host-range expression vector from Ptac; lacIq Apr | (5) |
| pEXG2-lrs1 | pEXG2 with lrs1 fragment from +1 to +191 | This study |
| pEXG2-rhlR | pEXG2 with rhlR allele constructed by SOE | This study |
| pLasR | pJTT201, pMMB67EH with lasR gene | (6) |
| pRhlR | pJTT202, pMMB67EH with rhlR gene | (6) |
| pET29a+Hfq | pET29a(+) with hfq-His6 in NdeI/XhoI site | This study |
| pCTX-pqsA-lacZ | Mini-CTX-lacZ with pqsA fragment (-246 to +231) in HindIII/XmaI site | This study |

**REFERENCES**

1. Liberati NT, Urbach JM, Miyata S, Lee DG, Drenkard E, et al. (2006) An ordered, nonredundant library of *Pseudomonas aeruginosa* strain PA14 transposon insertion mutants. *Proc Natl Acad Sci U S A* 103: 2833-2838.

2. Grant SG, Jessee J, Bloom FR, Hanahan D (1990) Differential plasmid rescue from transgenic mouse DNAs into *Escherichia coli* methylation-restriction mutants. *Proc Natl Acad Sci U S A* 87: 4645-4649.

3. Taylor RK, Manoil C, Mekalanos JJ (1989) Broad-host-range vectors for delivery of TnphoA: use in genetic analysis of secreted virulence determinants of *Vibrio cholerae*. *J Bacteriol* 171: 1870-1878.

4. Rietsch A, Vallet-Gely I, Dove SL, Mekalanos JJ (2005) ExsE, a secreted regulator of type III secretion genes in *Pseudomonas aeruginosa*. *Proc Natl Acad Sci U S A* 102: 8006-8011.

5. Furste JP, Pansegrau W, Frank R, Blocker H, Scholz P, et al. (1986) Molecular cloning of the plasmid RP4 primase region in a multi-host-range tacP expression vector. *Gene* 48: 119-131.

6. Thaden JT, Lory S, Gardner TS (2010) Quorum-sensing regulation of a copper toxicity system in *Pseudomonas aeruginosa*. *J Bacteriol* 192: 2557-2568.
